# Transcriptomic analysis of mammary gland tissues in lactating and non-lactating dairy goats reveals miRNA-mediated regulation of lactation, involution, and remodeling

**DOI:** 10.3389/fcell.2025.1604855

**Published:** 2025-05-30

**Authors:** Yanan Peng, Xinhua Duan, Linfan Zhang, Yiyi Guo, Jinlin Cao, Weiping Ao, Rong Xuan

**Affiliations:** ^1^ Key Laboratory of Livestock and Forage Resources Utilization around Tarim, Ministry of Agriculture and Rural Affairs, College of Animal Science and Technology, Tarim University, Alar, Xinjiang, China; ^2^ Animal Husbandry and Veterinary Bureau of Tashkurgan County, Kashgar, Xinjiang, China

**Keywords:** dairy goat, miRNA, lactation, mammary gland involution, mammary cells remodeling

## Abstract

**Background:**

Dynamic changes in the mammary gland during lactation and the dry period involve proliferation, secretion, apoptosis, and remodeling of mammary epithelial cells. MicroRNAs (miRNAs) are recognized as critical regulators of mammary gland development and lactation. However, their expression patterns and regulatory mechanisms at different lactation stages—particularly during mammary involution and remodeling—remain poorly understood in dairy goats.

**Methods:**

In this study, high-throughput sequencing was employed to analyze miRNA expression profiles in goat mammary tissues at five key stages: late gestation (LG), early lactation (EL), peak lactation (PL), late lactation (LL), and the dry period (DP). Differential expression analysis, miRNA clustering, Gene Ontology (GO) annotation, and Kyoto Encyclopedia of Genes and Genomes (KEGG) pathway enrichment were performed to predict the functions of target genes. A miRNA-mRNA regulatory network associated with mammary gland development was constructed, and functional validation experiments were conducted to confirm key regulatory relationships.

**Results:**

A total of 1,120 miRNAs were identified, including 408 known and 712 newly predicted miRNAs. Among them, 383 were significantly differentially expressed, with the largest number observed between the dry period and late gestation. Six expression-specific miRNA clusters were identified. Functional enrichment analysis indicated that these miRNAs may regulate epithelial cell proliferation, apoptosis, and tissue remodeling by targeting pathways such as energy metabolism, cell adhesion, and the PI3K/Akt signaling pathway. IGF1R was identified as a key regulatory gene in the miRNA-mRNA network related to mammary gland development. Experimental validation showed that chi-miR-423-3p inhibited mammary epithelial cell proliferation, induced G1/S cell cycle arrest, and promoted apoptosis by targeting IGF1R and suppressing the PI3K/Akt pathway.

**Conclusion:**

This study highlights the dynamic regulatory roles of miRNAs in the goat mammary gland across lactation stages. Notably, the miR-423-3p/IGF1R axis is a key regulator of mammary remodeling during the dry period, offering new insights into the molecular basis of mammary gland functional transitions.

## 1 Introduction

Lactation is a critical physiological process essential for mammary gland development and function in mammals, involving complex molecular regulatory mechanisms, including proliferation, differentiation, secretion, and apoptosis of mammary epithelial cells (Zhao et al., 2019; Hannan et al., 2023). Dairy goats (*Capra hircus*), as important dairy livestock, undergo significant morphological and functional changes in their mammary tissues during lactating and non-lactating (dry) periods (Cvek et al., 1998; Jiang et al., 2022; Xuan et al., 2022). During lactation, mammary epithelial cells proliferate extensively and synthesize milk proteins, lactose, and lipids to maintain continuous milk secretion (Hannan et al., 2023). In contrast, during the dry period, the mammary gland undergoes involution and remodeling, characterized by apoptosis of mammary epithelial cells, matrix remodeling, and macrophage-mediated phagocytosis, laying the foundation for the initiation of the subsequent lactation cycle (Zwick et al., 2018; Jena et al., 2019). The transition of the mammary gland through different lactation stages (late gestation, early lactation, peak lactation, late lactation, and dry period) requires precise molecular and cellular regulation (Fan et al., 2021). In dairy animals, a comprehensive understanding of the mechanisms governing mammary development and remodeling not only contributes to improved lactation efficiency but also significantly enhances the functional longevity of the mammary gland (Wu et al., 2022). However, the molecular mechanisms underlying mammary gland development and remodeling remain incompletely understood, particularly the regulatory roles played by microRNAs (miRNAs), which merit further investigation.

miRNAs are a class of small non-coding RNA molecules approximately 18–25 nucleotides (nt) in length, which regulate numerous physiological processes by binding to the 3′untranslated regions (3′UTR) of messenger RNAs (mRNAs), resulting in mRNA degradation or translational repression (Jin et al., 2013). Accumulating evidence indicates that miRNAs play essential roles in mammary gland development, lactation, and involution. For example, chi-miR-143-3p promotes mammary epithelial cell apoptosis in dairy goats by targeting Ndfip1 (Ji et al., 2019b); Bta-miR-200a regulates milk fat biosynthesis in bovine mammary epithelial cells by inhibiting the PI3K/Akt signaling pathway through targeting IRS2 (Tan et al., 2024); miR-205 has been associated with mammary stem cell properties, and miR-205-deficient mice exhibit enhanced mammary gland development (Cataldo et al., 2023). Furthermore, miRNA-342 and miR-206 play important roles in mammary epithelial cell differentiation and milk synthesis (Weng et al., 2016; Wang J. et al., 2019), whereas miR-424 and chi-miR-3880 regulate apoptosis and inflammatory responses during mammary involution (Wang F. et al., 2019; Zhang et al., 2023). Although several studies have sequenced and characterized miRNAs in dairy goat mammary tissues at different lactation or dry-period stages—for instance, constructing two small RNA libraries from goat mammary tissues at peak lactation and the dry period and identifying 346 conserved miRNAs and 95 novel miRNAs, performing sequencing analyses of goat mammary tissues at various lactation stages (Ji et al., 2012; Mobuchon et al., 2015; Hou et al., 2017; Ji et al., 2017), and analyzing miRNA expression profiles at different stages of the dry period (Li et al., 2012; Jiang et al., 2022)—these studies predominantly focus on specific time points or stages, lacking a systematic and comprehensive analysis of miRNA regulatory mechanisms throughout the entire lactation cycle (encompassing both lactation and dry periods).

In recent years, transcriptome sequencing (RNA-seq) has become a powerful tool for studying gene expression regulation, systematically revealing dynamic changes in transcriptomes under different physiological conditions. The integration of miRNA sequencing (miRNA-seq) with mRNA transcriptome data analysis allows for the identification of differentially expressed miRNAs and their potential target genes. This combined approach has been applied in mammary gland studies of various animal species, including dairy goats (Yu S. et al., 2017; Xuan et al., 2023), sheep (Zandi et al., 2020), cows (Zeng et al., 2023), and donkeys (Fei et al., 2022), providing insights into the miRNA regulatory networks involved in lactation, involution, and remodeling processes. Therefore, this study aims to systematically characterize the dynamic expression profiles and potential regulatory mechanisms of miRNAs in dairy goat mammary tissues throughout the entire lactation cycle (including lactating and dry periods), and to construct regulatory networks of key miRNAs and their target genes, elucidating their functional roles in mammary gland development, lactation maintenance, involution, and remodeling. Small RNA sequencing and omics analyses of mammary tissues collected at critical stages—late gestation, early lactation, peak lactation, late lactation, and the dry period—combined with target gene prediction and functional annotation, will be used to comprehensively depict the temporal regulatory networks of miRNAs controlling mammary gland structure and function. The findings will enhance the understanding of miRNA-mediated regulatory mechanisms underlying mammary gland development and function in dairy goats, providing novel molecular targets for improving lactation efficiency and mammary gland health.

## 2 Materials and methods

### 2.1 Ethics statement

Two datasets were used in this study, each including miRNA sequencing (miRNA-seq) and mRNA transcriptome sequencing (RNA-seq) data. The first dataset was approved and supervised by the Animal Protection and Ethics Committee of Shandong Agricultural University (protocol number: SDAUA-2018–048) (Xuan et al., 2020). The second dataset was approved by the Animal Experimental Ethics Committee (Institutional Animal Care and Use Committee) of Qingdao Agricultural University (Yu S. et al., 2017).

### 2.2 Experimental animals and mammary tissue sample collection

In this study, a total of 15 samples from two goat mammary gland transcriptome datasets were analyzed (Supplementary Table S1), covering five developmental stages: late gestation, early lactation, peak lactation, late lactation, and the dry period, with three biological replicates for each stage. The first dataset included nine non-lactating mammary tissue samples obtained from previous research conducted during my doctoral studies. These samples came from nine healthy Laoshan dairy goats raised under uniform conditions at Qingdao Aote Farm (Qingdao, Shandong Province, China), aged between 3 years and 8 months to 4 years and 1 month, and in their third parity. Mammary tissues were collected following slaughter during late gestation (LG; n = 3, 140 days post-mating), late lactation (LL; n = 3, 240 days *postpartum*), and the dry period (DP; n = 3, 300 days *postpartum*). The tissue collection procedure was as follows: after intravenous injection of sodium pentobarbital (100 mg/kg), muscle relaxation occurred, followed by cessation of heartbeat and respiration. The animal was then rapidly dissected, and mammary gland tissue was collected from the same side of the udder. Total RNA was extracted using TRIzol™ reagent (Invitrogen, Carlsbad, CA) and analyzed for quality using an Agilent 2,100 Bioanalyzer (Agilent Technologies, Waldbronn, Germany), with only samples having RNA integrity values greater than 8 included in further analyses. Small RNA and mRNA sequencing libraries were prepared using the NEBNext^®^ Ultra™ RNA Library Prep Kit and TruSeq RNA Library Prep Kit v2 (Illumina, San Diego, United States), respectively, and sequenced on Illumina/Solexa and HiSeq 2,500 platforms. Data were deposited in the NCBI GEO database under accession numbers GSE243232 and GSE185981. The second dataset comprised mammary gland tissues collected from 2-year-old Laoshan dairy goats during early lactation (5 days *postpartum*) and peak lactation (30 days *postpartum*), with small RNA sequencing performed on the Illumina-HiSeq X Ten platform and mRNA sequencing conducted on the Illumina HiSeq 2,500 platform. Raw data were submitted to the NCBI SRA database with accession number PRJNA361394.

### 2.3 Quality control of sequencing data and alignment to the reference genome

Quality control of all samples was performed using FastQC software (version 0.11.9, Babraham Bioinformatics, Cambridge, United Kingdom) (Andrews, 2010), and high-throughput sequencing reads were processed with Trimmomatic software (version 0.39, USADEL Laboratory, Jülich, Germany) to remove adapter sequences, primers, poly-A tails, and low-quality reads (Bolger et al., 2014). The goat reference genome index (NCBI RefSeq assembly: GCF_001704415.2) was built using bowtie-build. The sequencing data were aligned to the goat reference genome using miRDeep2 software (Version 2.0.1.1, N. Rajewsky Lab, Berlin, Germany) (Friedländer et al., 2012) to identify known and novel miRNAs. Mature and precursor miRNA sequences required for alignment were obtained from the miRBase database (Kozomara et al., 2019). Newly identified miRNAs were merged with known miRNAs to create a comprehensive annotation reference file, and microRNA quantification was performed using the quantifier.pl script from the miRDeep2 package.

### 2.4 Identification of differentially expressed miRNAs

Low-expression miRNAs were filtered based on an expression threshold of FPKM ≥0.5. The miRNA family classifications were annotated according to the miRBase database. The distribution of miRNA lengths was visualized using the hist() function in R software (version 4.4.3). Principal component analysis (PCA) and relative log expression (RLE) analysis of mammary gland samples were performed using the RUVSeq package (version 1.40.0). Differential expression analysis was conducted using the edgeR package (version 4.4.2) across ten comparative groups: EL vs. LG, PL vs. LG, LL vs. LG, DP vs. LG, PL vs. EL, LL vs. EL, DP vs. EL, LL vs. PL, DP vs. PL, and DP vs. LL. miRNAs were considered differentially expressed if they satisfied the criteria of |log2Fold Change| > 1 and false discovery rate (FDR) < 0.05. The R package UpSetR (version 1.4.0) was used to generate Venn diagrams to illustrate the overlap of differentially expressed miRNAs among comparison groups. Additionally, a heatmap depicting the expression profiles of all differentially expressed miRNAs was generated using the pheatmap package (version 1.0.12) in R.

### 2.5 Temporal profiling of miRNA expression

To further investigate the expression patterns of differentially expressed miRNAs across five developmental stages in mammary gland tissues, we performed clustering analysis of these miRNAs using the c-means method implemented in the TCseq package (version 1.30.0). Additionally, we plotted line charts illustrating the expression levels of distinct miRNAs across five developmental stages.

### 2.6 Prediction of miRNA target genes and functional enrichment analysis

The miRanda software (version 3.3a) was used to predict the targeting relationships between miRNAs and differentially expressed mRNAs, with an energy threshold set at −10 and a score threshold set at 150 (Enright et al., 2003). The R package psych (version 2.2.3) was employed to calculate Pearson correlations between differentially expressed miRNAs and protein-coding genes, followed by significance testing. Protein-coding genes were filtered based on an absolute correlation value ≥0.5 and a *P*-value <0.05. Finally, the intersection of structural prediction results and expression correlation analyses was determined as the definitive miRNA-mRNA targeting relationships. The clusterProfiler package (version 4.12.6) was utilized to perform GO annotation and KEGG pathway enrichment analyses for genes within each cluster exhibiting miRNA-mRNA interactions (Wu et al., 2021). Bar plots and dot plots were generated to visualize the GO and KEGG enrichment results, respectively.

### 2.7 Construction of miRNA–target gene regulatory network

Based on the results of GO and KEGG analyses, potential target genes and their corresponding miRNAs associated with substance transport and synthesis, mammary gland development, cell growth and differentiation, lactation regulation, and apoptosis were selected. Cytoscape software (version 3.7.1) was used to construct a regulatory network between differentially expressed miRNAs and their target genes. Protein-protein interaction analysis was further performed on the target genes within the miRNA-mRNA network, and hub genes were identified using a degree threshold of ≥7. These hub genes and their regulatory miRNAs were visualized using Cytoscape software (Shannon et al., 2003). In addition, GO functional annotation and KEGG pathway enrichment analyses were conducted on the genes in the regulatory network to uncover the potential functions of miRNAs and their target genes, as well as their involvement in key regulatory pathways. The above analysis will provide theoretical support and experimental candidate targets for the subsequent validation of specific miRNA functions at the cellular level.

### 2.8 Culture of mammary epithelial cells

Mammary tissue was surgically collected from late-gestation goats and rinsed with sterile phosphate-buffered saline (PBS) to remove blood and debris. Adipose and connective tissues were carefully excised using ophthalmic scissors, and the remaining tissue was minced into small fragments (0.5–1 mm^3^). Primary goat mammary epithelial cells (GMECs) were cultured using a tissue explant method previously established in our laboratory. The cells were maintained in Dulbecco’s Modified Eagle Medium/Nutrient Mixture F-12 (DMEM/F-12) supplemented with 10% fetal bovine serum (FBS), 0.25 mM hydrocortisone, 5 μg/mL insulin (Sigma), 50 U/mL penicillin-streptomycin, and 10 ng/mL epidermal growth factor (EGF). Cultures were incubated at 37°C in a humidified atmosphere containing 5% CO_2_. GMECs were seeded into 6-well culture plates at a density of 3 × 10^5^ cells per well, and transfection experiments were conducted when the cells reached approximately 75% confluence.

### 2.9 Dual-luciferase reporter assay

Prior to validating the targeting relationship, chi-miR-423-3p and chi-miR-345-3p were individually overexpressed in primary goat mammary epithelial cells to evaluate their effects on the expression of the predicted target gene *IGF1R*. Subsequently, the interaction between chi-miR-423-3p and the 3′-untranslated region (3′UTR) of *IGF1R* (XM_018065947.1) was predicted using the miRanda software (version 3.3a). Candidate binding sites with a minimum free energy (MFE) ≤ −15 kcal/mol and a score ≥100 were selected. Primers containing restriction enzyme sites were designed to amplify the 3′UTR fragment of *IGF1R* by reverse transcription PCR (RT-PCR). The PCR product was digested with *Nhe I* and *Xba I* and cloned into the pmirGLO dual-luciferase reporter vector (Promega). Primary goat mammary epithelial cells were used for subsequent transfection experiments, with four groups established: the control group (Control) containing the empty pmirGLO vector; the wild-type group (WT) containing the native 3′UTR sequence of *IGF1R*; the mutant group (MT) with mutations in the predicted miRNA binding site; and a positive control group (miRNA_Positive) containing the reverse complement sequence of the miRNA. Dual-luciferase reporter assays were performed in 24-well plates, with each well receiving 300 ng of reporter construct and 30 pmol of miRNA mimic, co-transfected using Lipofectamine 3,000 (Invitrogen). Each condition was set up in triplicate. After 48 h, firefly and Renilla luciferase activities were measured using the Dual-Luciferase^®^ Reporter Assay System (Promega) on a GloMax^®^ 20/20 luminometer (Promega).

### 2.10 Analysis of cell apoptosis and cell cycle

Goat mammary epithelial cells were seeded in 6-well plates at a density of 3 × 10^5^ cells per well. At 48 h post-transfection, 5 μL of Annexin V-FITC and 10 μL of propidium iodide (PI) staining solution (Invitrogen) were added to each well, followed by incubation in the dark at room temperature for 10 min. Cell apoptosis and cell cycle analysis were subsequently performed using a BD flow cytometer (BD Biosciences). Flow cytometry data were analyzed with FlowJo software version 7.6.1 (BD Biosciences). In addition, Western blotting was conducted to assess the protein expression levels of caspase-3, Bax, and BCL-2 in cells collected 48 h after transfection.

### 2.11 Assessment of cell proliferation

Goat mammary epithelial cells were seeded into 96-well plates at a density of 8,000 cells per well. When cell confluency reached approximately 75%, transfection experiments were performed. Cell proliferation was assessed using the CCK-8 assay. Specifically, cells were harvested at 0, 24, 48, and 72 h post-transfection. At each time point, 10 μL of CCK-8 solution (Beyotime) was added to each well, followed by incubation at 37°C for 1 h. The absorbance at 450 nm was measured using a SpectraMax ID3 microplate reader (Molecular Devices). Line graphs representing cell proliferation activity were generated using GraphPad Prism software (version 8).

### 2.12 Western blot analysis

Total protein was extracted from mammary epithelial cells using RIPA lysis buffer (Beyotime). Protein concentrations were quantified using the bicinchoninic acid (BCA) assay (Beyotime) on a microplate reader (Molecular Devices) at 562 nm. Equal amounts of protein (20 μg per sample) were separated by Sodium Dodecyl Sulfate Polyacrylamide Gel Electrophoresis (SDS-PAGE) using a 10% separating gel and a 5% stacking gel (Beyotime), and subsequently transferred onto polyvinylidene difluoride (PVDF) membranes. Membranes were blocked in 5% skim milk for 1.5 h, followed by incubation with primary antibodies specific to the target proteins at 4°C overnight. After three washes with TBST (1 × Tris-buffered saline containing Tween 20), membranes were incubated with horseradish peroxidase (HRP)-conjugated secondary antibodies at room temperature for 50 min. Immunoreactive bands were visualized using enhanced chemiluminescence (ECL) reagent (Beyotime) and imaged with the Azure 300 chemiluminescence imaging system (Azure Biosystems). Protein expression levels were quantified using ImageJ software (version 1.48; NIH), with GAPDH serving as the internal loading control. Western blot images were first converted to 8-bit grayscale format, and background noise was eliminated through background subtraction to improve measurement accuracy. The measurement settings were configured to record parameters such as area, mean gray value, minimum and maximum gray values, and integrated density. The image scale was calibrated using pixels as the unit of measurement, and the images were then inverted to enhance the visibility of protein bands, allowing clear identification of each signal. Identical-sized rectangular regions were manually selected around each band to ensure consistency across measurements, and the integrated density of each band was determined. The relative expression of the target protein was calculated by normalizing its band intensity to that of the corresponding GAPDH band. Each sample was analyzed in triplicate, and the average value was used for subsequent statistical analysis. The antibodies used and their dilutions were as follows: anti-IGF1R (1:1,000, ab131487, Abcam), anti-p21 (1:1,000, ab109199, Abcam), anti-p53 (1:1,000, ab26, Abcam), anti-IRS1 (1:1,000, ab313437, Abcam), anti-CCND1 (1:1,500, #55506, CST), anti-GAPDH (1:2000, #2118, CST), anti-PI3K (1:1,000, #4257, CST), anti-p-PI3K (1:1,000, #17366, CST), anti-Akt (1:1,000, #4691, CST), anti-p-Akt (1:1,000, #4060, CST), anti-caspase-3 (1:2000, #14220, CST), anti-BCL-2 (1:1,000, ab182858, Abcam), anti-Bax (1:1,000, #2772, CST), and HRP-conjugated goat anti-rabbit IgG (1:2000, CW0103, CWBIO).

### 2.13 Real-time quantitative reverse transcription PCR (RT-qPCR)

Primers for genes and miRNAs were designed using NCBI Primer-BLAST and miRprimer software, respectively. Total RNA was isolated from mammary tissue using the Trizol method. Quantification of mRNA and miRNA was performed according to the instructions of the One Step TB Green^®^ PrimeScript™ RT-PCR Kit (Perfect Real Time) (Takara) and the Mir-X™ miRNA qRT-PCR TB Green™ Kit (Clontech), respectively. Reactions were carried out using the LightCycler^®^ 96 System (Roche). Primer amplification efficiencies were calculated by generating standard curves. The geometric mean of two reference genes (*GAPDH* and *MRPL39*), previously validated in related studies (Xuan et al., 2022), was used as a reliable normalization factor. Relative gene expression levels were calculated using a modified Pfaffl method (Pfaffl, 2001) (for detailed calculation steps, see: https://toptipbio.com/qpcr-multiple-reference-genes/). Primer sequences and their efficiencies are listed in Supplementary Table S2.

### 2.14 Statistical analysis

The Student’s t-test was used to test the significance of the difference between the experimental group and the control group; a *P* -value <0.05 indicated a significant difference.

## 3 Results

### 3.1 Basic statistical analysis of sequencing results

**FIGURE 1:**
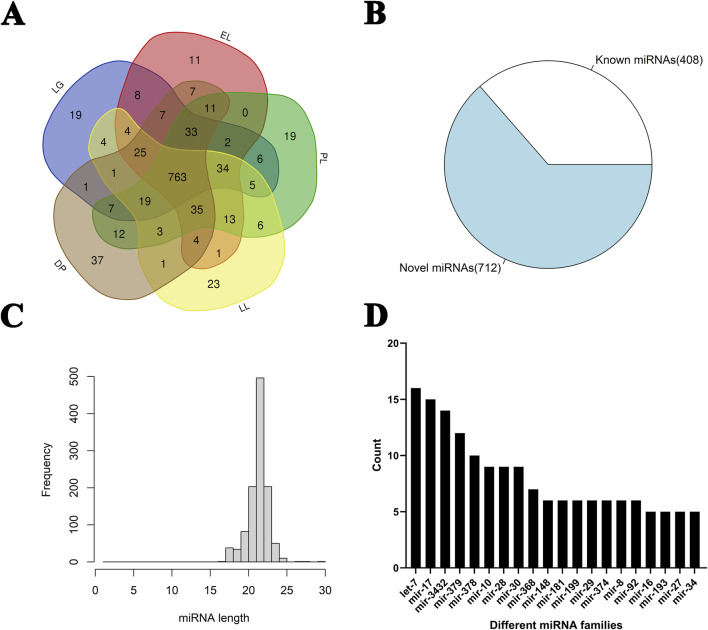
Identification and classification of miRNAs in goat mammary glands at different stages of the lactation cycle. **(A)** Venn diagram showing the distribution of identified miRNAs among five different stages of the lactation cycle in goat mammary glands: late gestation (LG), early lactation (EL), peak lactation (PL), late lactation (LL), and dry period (DP). **(B)** Pie chart showing the number of known and novel miRNAs identified across all samples. A total of 1,120 miRNAs were identified, including 408 known miRNAs and 712 novel miRNAs. **(C)** Length distribution of the identified miRNAs. Most miRNAs were 21–23 nucleotides in length, with 22 nt being the most frequent. **(D)** Bar chart displaying the number of members in the top 20 most abundant miRNA families. The let-7, mir-17, mir-3432, and mir-378 families showed high abundance across different lactation stages.

A total of 311,337,713 reads were obtained from 15 small RNA libraries through miRNA sequencing. After filtering out low-quality reads, 291,099,958 clean reads were retained (Supplementary Table S1). The average mapping rate across libraries was 89%, with the lowest mapping rate ≥87%. In total, 1,120 miRNAs were identified, including 408 known and 712 novel miRNAs (Supplementary Table S3). Among these, 763 miRNAs were expressed across all five developmental stages (Figure 1A). The highest number of expressed miRNAs (967) was observed at the peak lactation stage, while the lowest number was detected in the late gestation stage. The miRNA length distribution ranged from 20 to 23 nucleotides, with 22-nt miRNAs being the most abundant (Figure 1C). Family analysis revealed that miRNAs from families such as let-7, mir-17, mir-3432, and mir-379 were particularly enriched (Figure 1D).

### 3.2 Identification of differentially expressed miRNAs

**FIGURE 2:**
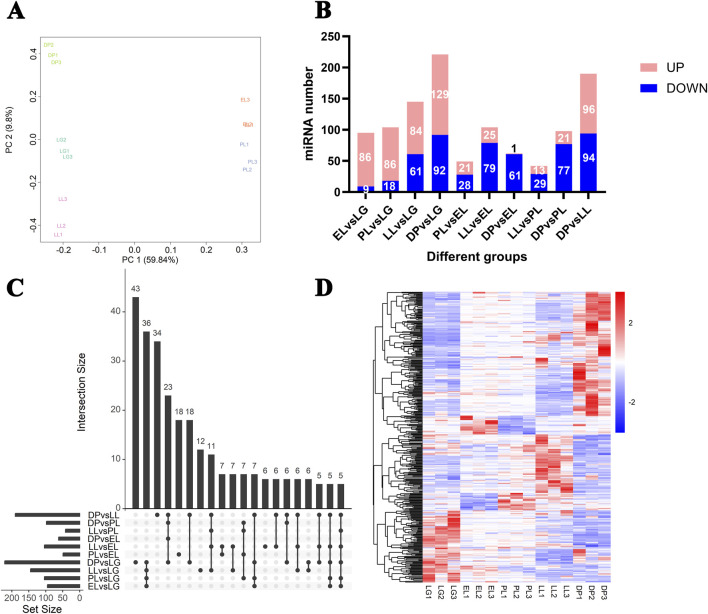
Analysis of differentially expressed miRNAs in goat mammary glands at different stages of the lactation cycle. **(A)** Principal component analysis (PCA) of miRNA expression profiles from mammary gland tissues at different physiological stages, showing clear separation among five stages. **(B)** Bar plot showing the number of differentially expressed (DE) miRNAs in pairwise comparisons between different physiological stages. Red bars indicate upregulated miRNAs, and blue bars indicate downregulated miRNAs. **(C)** UpSet plot showing the intersection of differentially expressed miRNAs among all pairwise comparisons. The vertical bars represent the number of DE miRNAs shared by the groups connected by black dots, and the horizontal bars represent the total number of DE miRNAs in each comparison. **(D)** Hierarchical clustering heatmap of the all differentially expressed miRNAs across all samples. Each row represents one miRNA, and each column represents one biological replicate from different physiological stages. Red indicates high expression, and blue indicates low expression.

Principal component analysis of miRNA expression profiles across all samples showed that samples from the same developmental stage clustered together (Figure 2A). A total of 383 differentially expressed miRNAs (DE-miRNAs) were identified (Figure 2B; Supplementary Table S4). The largest number of DE-miRNAs was observed in the comparison between the DP and LG, with 129 miRNAs upregulated and 92 downregulated in the DP. The fewest DE-miRNAs were detected in the comparison between LL and PL.

Notably, EL vs. LG, DP vs. LL, and DP vs. LG represent three key transitions: lactation initiation, lactation cessation, and the onset of mammary gland remodeling, respectively. In the comparison between EL and LG (Supplementary Table S4), several known miRNAs, including chi-miR-1, chi-miR-3432-5p, chi-miR-223-5p, chi-miR-708-5p, chi-miR-146b-3p, and chi-miR-34c-5p, were significantly upregulated. In addition, novel miRNAs NC_030826.1_25425, NC_030815.1_12105, and NC_030828.1_27534 were highly upregulated with fold changes greater than 128. Conversely, known miRNAs chi-miR-30f-3p, chi-miR-30f-5p and novel miRNAs NC_030808.1_1640, NC_030809.1_1828 were significantly downregulated.

In the comparison between the DP and LL, several known miRNAs including chi-miR-99a-5p, chi-miR-206, chi-miR-218, chi-miR-147-5p, chi-miR-105a, and chi-miR-34b-3p, as well as novel miRNAs NC_030828.1_27139, NC_030820.1_17538, and NC_030823.1_21668, were upregulated during the DP with fold changes greater than 16. On the other hand, known miRNAs chi-miR-148a-3p, chi-miR-30f-3p, chi-miR-146b-3p, chi-miR-122, and novel miRNAs NC_030828.1_27538, NC_030828.1_27537, and NC_030812.1_7001 were downregulated in the DP, with fold changes exceeding 256.

In the comparison between the DP and LG, known miRNAs chi-miR-1, chi-miR-3432-5p, chi-miR-708-5p, chi-miR-34c-5p, and chi-miR-29b-3p, along with novel miRNAs NC_030815.1_12105, NC_030828.1_27534, and NC_030831.1_30149, were significantly upregulated during the DP with fold changes greater than 128. Conversely, chi-miR-148a-3p, chi-miR-30f-3p, chi-miR-381, chi-miR-30f-5p, and novel miRNAs NC_030835.1_32988, NC_030811.1_6576, and NC_030826.1_25423 were significantly downregulated, with fold changes greater than 14.

An UpSet plot (Figure 2C) showed the distribution of DE-miRNAs across different comparison groups, and a heatmap (Figure 2D) illustrated the dynamic expression changes of the 383 DE-miRNAs across the five developmental stages.

### 3.3 Target genes prediction and expression analysis of differentially expressed miRNAs

**FIGURE 3:**
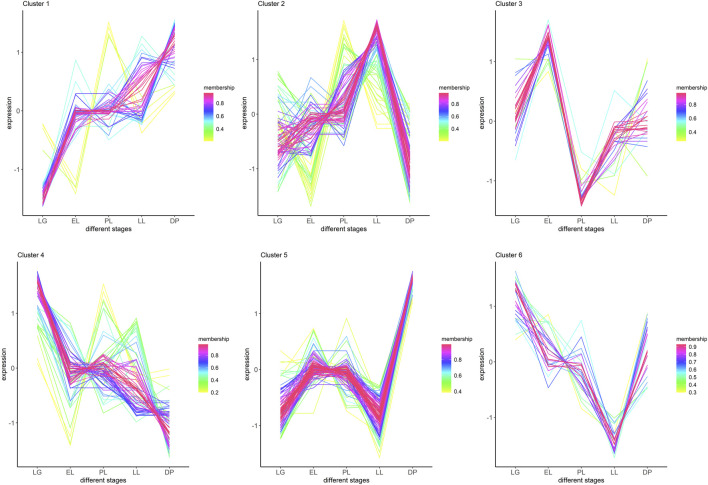
Clustering of differentially expressed miRNAs based on their expression patterns across lactation cycle stages. Differentially expressed miRNAs were grouped into six clusters using time-series expression pattern analysis. The x-axis represents different physiological stages: late gestation (LG), early lactation (EL), peak lactation (PL), late lactation (LL), and dry period (DP). The y-axis indicates standardized expression levels (Z-score). Each colored line represents an individual miRNA, with color intensity indicating the membership value (confidence of assignment to the cluster). Clusters show distinct temporal expression trends across lactation cycle stages, suggesting stage-specific regulatory roles for different miRNA subsets.

Using the miRanda software, a total of 2,121,562 miRNA–mRNA structural targeting relationships were predicted (score ≥150, energy ≤ −15), while correlation analysis identified 243,723 miRNA–mRNA quantitative targeting relationships (*P* < 0.05, |correlation| ≥ 0.5). The intersection of these two methods yielded 33,283 high-confidence miRNA–mRNA pairs (Supplementary Table S5). Clustering analysis of DE-miRNAs across various physiological stages identified six distinct expression patterns (Figure 3; Supplementary Table S6). Specifically, Cluster 1 (20 miRNAs) and Cluster 5 (108 miRNAs) showed high expression levels during the dry period (DP); Cluster 2 (94 miRNAs) was predominantly expressed in late lactation (LL); Cluster 3 (29 miRNAs) exhibited a marked upregulation during early lactation (EL), while Cluster 4 (74 miRNAs) and Cluster 6 (28 miRNAs) showed elevated expression in late gestation (LG). These findings highlight the stage-specific expression characteristics of miRNAs in mammary gland tissue.

### 3.4 Functional enrichment analysis of miRNA target genes

**FIGURE 4:**
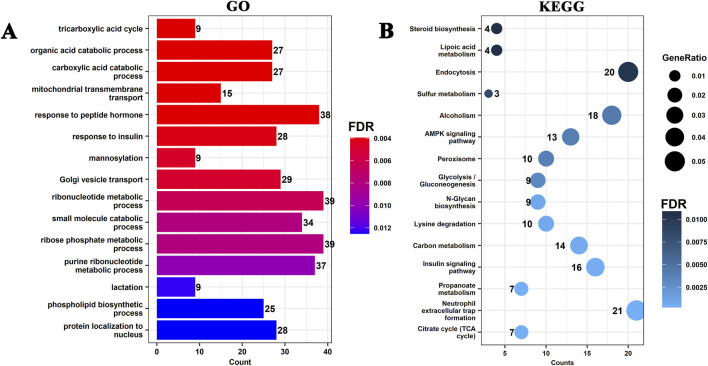
Functional enrichment analysis of target genes of differentially expressed miRNAs in Cluster 1. **(A)** Gene Ontology (GO) enrichment analysis of target genes associated with Cluster 1 miRNAs. The top enriched GO terms (biological processes) are shown, with the x-axis representing the number of genes (Count) and the color indicating the significance level (FDR). **(B)** Kyoto Encyclopedia of Genes and Genomes (KEGG) pathway enrichment analysis of Cluster 1 target genes. The size of the dots represents the GeneRatio (ratio of target genes enriched in the pathway), and the color represents the FDR.

The molecular functions of target genes of DE-miRNAs were analyzed using GO, and their involvement in signaling pathways was investigated through KEGG pathway enrichment analysis to elucidate the major functions and regulatory pathways of DE miRNAs (Supplementary Figures S2–S6; Supplementary Table S7). miRNAs in Cluster 1 and Cluster 5 were specifically highly expressed during the DP. In Cluster 1 (Figure 4), GO terms related to substance metabolism, including tricarboxylic acid cycle, organic acid catabolic process, carboxylic acid catabolic process, mitochondrial transmembrane transport, and response to peptide hormone, were significantly enriched. KEGG pathways such as citrate cycle (TCA cycle), propanoate metabolism, carbon metabolism, lysine degradation, and N-Glycan biosynthesis, all related to substance synthesis and metabolism, were also significantly enriched.

In Cluster 5 (Supplementary Figure S5), GO terms such as sterol biosynthetic process, cholesterol biosynthetic process, secondary alcohol biosynthetic process, secondary alcohol metabolic process, small molecule catabolic process, and sterol metabolic process were significantly enriched. Enriched KEGG pathways included steroid biosynthesis, biosynthesis of amino acids, fatty acid metabolism, cysteine and methionine metabolism, terpenoid backbone biosynthesis, and protein export. These miRNAs may regulate energy metabolism, lipid synthesis, amino acid metabolism, and cell communication, thereby promoting metabolic remodeling and structural reorganization of the mammary gland during the DP, laying the foundation for regeneration in the subsequent lactation cycle.

miRNAs in Cluster 2 were specifically highly expressed in the LL (Supplementary Figure S2). GO terms such as ncRNA processing, nucleocytoplasmic transport, nuclear transport, histone modification, ribonucleoprotein complex biogenesis, and ribosome biogenesis were significantly enriched. KEGG pathways such as endocytosis, focal adhesion, proteoglycans in cancer, regulation of actin cytoskeleton, notch signaling pathway, and hepatitis B were also significantly enriched. These miRNAs may regulate nucleic acid metabolism, cytoskeleton remodeling, endocytosis, and Notch signaling in mammary epithelial cells, thus contributing to functional adjustments of the mammary gland during late lactation and providing a molecular regulatory mechanism for lactation cessation and mammary gland remodeling.

miRNAs in Cluster 3 were specifically highly expressed during the EL (Supplementary Figure S3). GO terms such as actin filament bundle, cluster of actin-based cell projections, ruffle membrane, and phagophore assembly site were significantly enriched. KEGG pathways including terpenoid backbone biosynthesis, autophagy–animal, AMPK signaling pathway, longevity regulating pathway, and spinocerebellar ataxia were also significantly enriched. These miRNAs may regulate cytoskeleton remodeling, autophagy, and energy metabolism in mammary epithelial cells, enhancing secretory function in early lactation and maintaining mammary gland homeostasis through hormone metabolism and longevity-related pathways to meet the high metabolic demands of the lactation period.

miRNAs in Cluster 4 and Cluster 6 were specifically highly expressed during the LG. In Cluster 4 (Supplementary Figure S5), GO terms such as cell-substrate adhesion, small GTPase mediated signal transduction, regulation of small GTPase mediated signal transduction, cell-matrix adhesion, and positive regulation of cell adhesion were significantly enriched. KEGG pathways such as focal adhesion, proteoglycans in cancer, regulation of actin cytoskeleton, PI3K-Akt signaling pathway, and ECM-receptor interaction were significantly enriched. In Cluster 6 (Supplementary Figure S6), miRNA target genes were significantly enriched in GO terms such as manganese ion transport, manganese ion transmembrane transport, transition metal ion transport, and carbohydrate transport. KEGG pathways including biosynthesis of amino acids, steroid biosynthesis, ferroptosis, adipocytokine signaling pathway, and fatty acid biosynthesis were significantly enriched. miRNAs in Cluster 4 may regulate cell adhesion, migration, and cytoskeleton remodeling of mammary epithelial cells, promoting the maturation of ducts and alveoli and preparing structurally for secretory function in lactation. miRNAs in Cluster 6 may regulate the metabolism of metal ions, carbohydrates, amino acids, and lipids, enhancing nutrient uptake and biosynthetic capacity of the mammary gland to ensure efficient metabolism and secretion during lactation.

### 3.5 Regulatory network analysis of key genes and miRNAs related to mammary gland development

**FIGURE 5:**
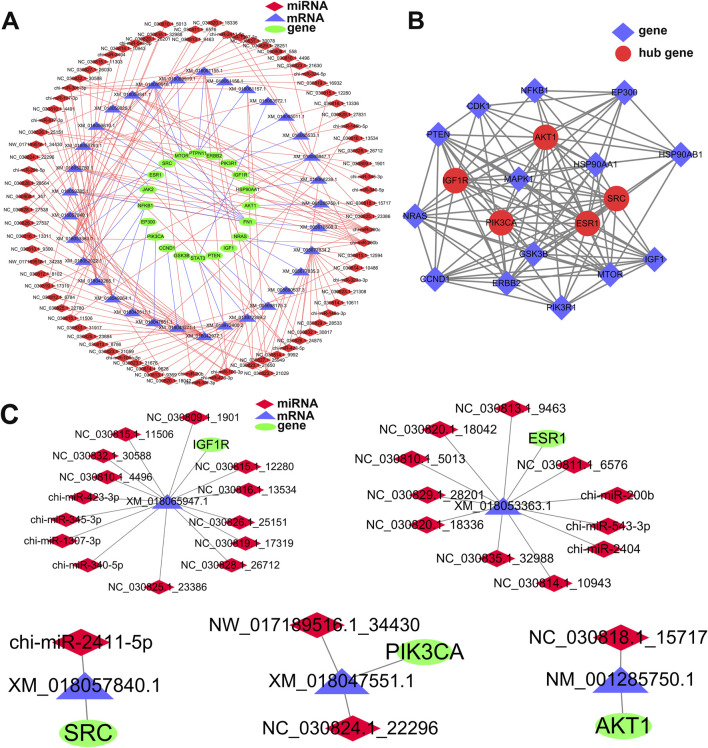
Regulatory and interaction networks of differentially expressed miRNAs and their target genes related to mammary gland development. **(A)** Regulatory network of differentially expressed miRNAs and their predicted target genes involved in mammary gland development. Red diamonds represent differentially expressed miRNAs, blue triangles represent mRNAs, and green ellipses represent target genes. Edges indicate regulatory relationships. **(B)** Protein-protein interaction (PPI) network analysis of miRNA target genes. Five hub genes (*AKT1*, *PIK3CA*, *IGF1R*, *ESR1*, and *SRC*) were identified based on their centrality in the network. Red circles represent hub genes, and blue diamonds represent other interacting genes. **(C)** Regulatory subnetwork showing the five hub genes and their associated differentially expressed miRNAs. Green ellipses indicate hub genes, red diamonds indicate miRNAs, and blue triangles indicate mRNAs.

**FIGURE 6:**
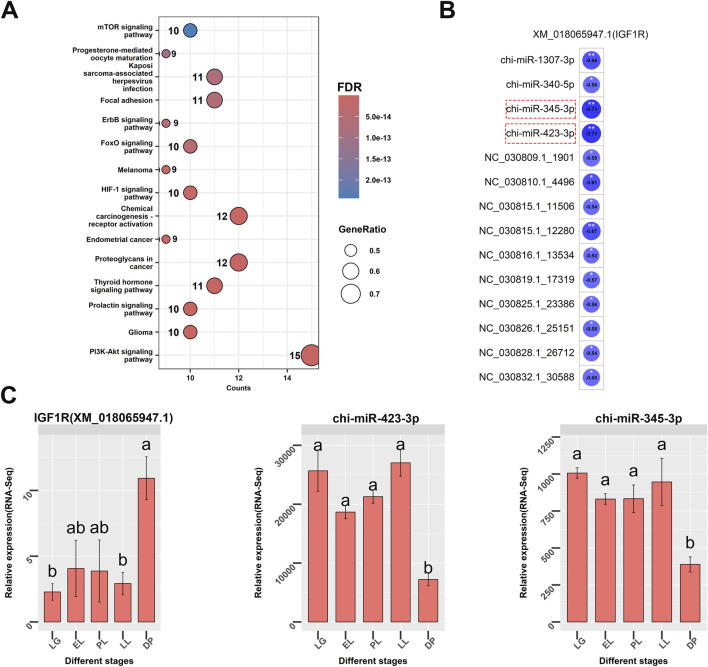
KEGG pathway enrichment analysis of genes related to mammary gland development and expression correlation analysis between IGF1R and its associated miRNAs. **(A)** KEGG enrichment analysis of genes associated with mammary gland development. Dot size represents the gene ratio, and color indicates the false discovery rate (FDR). **(B)** Regulatory network of IGF1R (XM_018065947.1) and its associated miRNAs. chi-miR-345-3p and chi-miR-423-3p, highlighted with red boxes, are closely related to IGF1R (correlation value = −0.73). **(C)** Relative expression levels of IGF1R (left), chi-miR-423-3p (middle), and chi-miR-345-3p (right) in goat mammary gland tissues at different physiological stages: late gestation (LG), early lactation (EL), peak lactation (PL), late lactation (LL), and dry period (DP). Different letters indicate statistically significant differences between stages (*P* < 0.05).

Based on GO and KEGG annotation of target genes, a regulatory network related to mammary gland development was constructed using Cytoscape, comprising 77 miRNAs, 20 genes, and 29 mRNAs (Figure 5A). In the PPI network (Figure 5B), five hub genes were identified: *IGF1R*, *ESR1*, *SRC*, *PIK3CA*, and *AKT1*. Bioinformatic predictions indicated that *IGF1R* is regulated by known miRNAs chi-miR-423-3p, chi-miR-345-3p, chi-miR-1307-3p, and chi-miR-340-5p, as well as novel miRNAs NC_030809.1_1901, NC_030810.1_4496, NC_030815.1_11506, and NC_030815.1_12280 (Figure 5C). *ESR1* is targeted by known miRNAs chi-miR-200b, chi-miR-543-3p, and chi-miR-2404, and also by novel miRNAs NC_030829.1_28201, NC_030835.1_32988, and NC_030810.1_5013. *SRC* is regulated by chi-miR-2411-5p; *PIK3CA* is targeted by NC_030824.1_22296 and NW_017189516.1_34430; *AKT1* is regulated by NC_030818.1_15717. Among the 20 genes, 15 were significantly enriched in the PI3K-Akt signaling pathway (Figure 6A), with *IGF1R* located upstream and specifically highly expressed during the dry period. Therefore, subsequent mammary epithelial cell experiments will focus on *IGF1R* and its regulatory miRNAs.

### 3.6 Targeted regulatory relationship between chi-miR-423-3p and *IGF1R*

**FIGURE 7:**
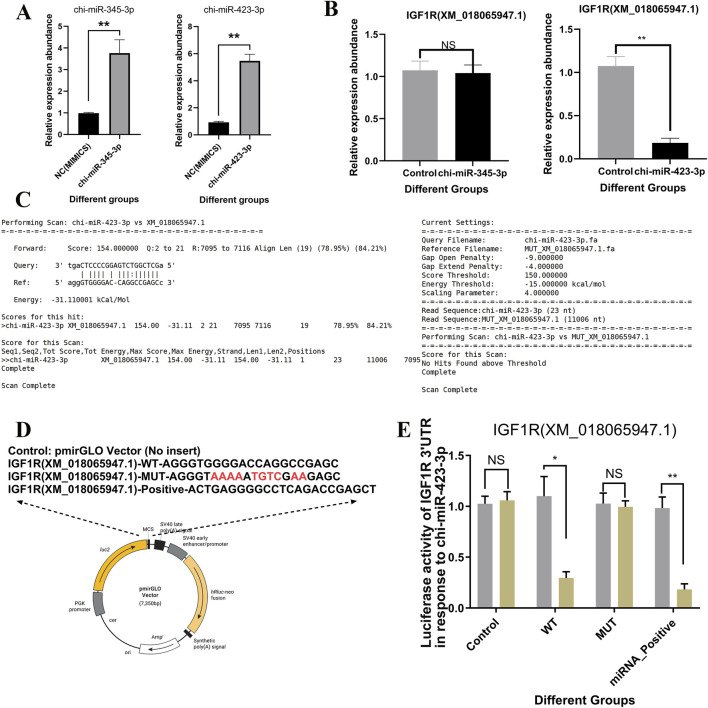
Validation of the targeting relationship between chi-miR-345-3p/chi-miR-423-3p and *IGF1R*. **(A)** Overexpression of chi-miR-345-3p and chi-miR-423-3p in goat primary mammary epithelial cells significantly increased the expression levels of the respective miRNAs, as determined by RT-qPCR. **(B)** Overexpression of chi-miR-423-3p significantly reduced the mRNA expression level of IGF1R (XM_018065947.1), whereas chi-miR-345-3p showed no significant effect. **(C)** The interaction between chi-miR-423-3p and the 3′UTR of IGF1R was predicted using miRanda software, showing strong base pairing and a minimum free energy of −31.1 kcal/mol. **(D)** Schematic representation of luciferase reporter constructs containing the wild-type (WT), mutant (MUT), and positive control (Positive) sequences of the IGF1R 3′UTR. The mutated seed region in the MUT construct is highlighted in red. **(E)** Dual-luciferase reporter assay in goat primary mammary epithelial cells showed that chi-miR-423-3p significantly suppressed luciferase activity in the WT and Positive groups, but not in the MUT group, confirming a direct targeting interaction between chi-miR-423-3p and the IGF1R 3′UTR. Data are presented as means ± SEM from three independent experiments. Data are presented as mean ± SEM. *P* < 0.05 (*), *P* < 0.01 (**), NS: not significant.

In this study, based on the expression correlation between miRNAs and *IGF1R*, two known miRNAs, chi-miR-345-3p and chi-miR-423-3p, were selected (Figure 6B). Both were lowly expressed during the dry period and showed a significant negative correlation with *IGF1R* expression. Overexpression of these two miRNAs in mammary epithelial cells revealed that chi-miR-423-3p significantly reduced the expression level of *IGF1R*, while chi-miR-345-3p had no effect (Figures 7A,B). Dual-luciferase reporter assay showed that (Figure 7E), compared with the control group, the luciferase activity of the wild-type *IGF1R* (*XM_018065947.1*) reporter vector was reduced by 73.11% ± 4.64% upon co-transfection with chi-miR-423-3p mimic, whereas the luciferase activity of the mutant *IGF1R* (*XM_018065947.1*) reporter vector remained unchanged. These results indicate that chi-miR-423-3p has a targeted regulatory relationship with *IGF1R*.

### 3.7 Regulation of IGF1R protein levels by *IGF1R* overexpression and miR-423-3p

**FIGURE 8:**
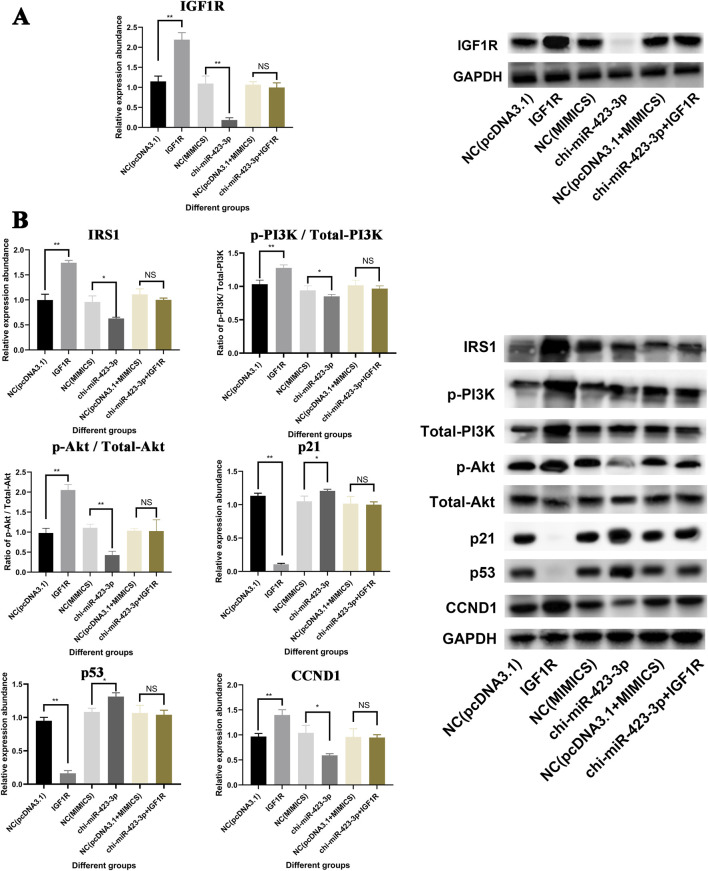
Regulation of the IGF1R-PI3K/Akt Signaling Pathway Mediated by chi-miR-423-3p. **(A)** chi-miR-423-3p suppresses IGF1R protein expression, and IGF1R overexpression rescues the inhibition. Western blot analysis and quantification of IGF1R expression levels in different treatment groups. GAPDH was used as a loading control. **(B)** Effects of chi-miR-423-3p and IGF1R on the PI3K/Akt signaling pathway. Western blot and quantification of key components in the IGF1R-PI3K/Akt signaling pathway, including IRS1, p-PI3K/Total-PI3K, p-Akt/Total-Akt, and downstream regulators p21, p53, and CCND1. Data are shown as mean ± SEM (n = 3). *P* < 0.05 (*), *P* < 0.01 (**), NS: not significant.

Overexpression of IGF1R in mammary epithelial cells significantly increased protein expression levels, reaching 1.91 ± 0.16 fold compared to the control group, as shown by Western blotting (Figure 8A). In contrast, overexpression of chi-miR-423-3p significantly suppressed IGF1R protein expression, with a marked reduction of 82.27% ± 8.66% compared to the control group (*P* < 0.01). Co-overexpression of IGF1R reversed the chi-miR-423-3p-mediated downregulation of IGF1R.

### 3.8 Regulation of the IGF1R–PI3K/Akt signaling pathway mediated by chi-miR-423-3p

Western blot analysis showed that, compared to the control group (Figure 8B), IGF1R overexpression significantly upregulated the expression of IRS1, the ratios of p-PI3K/Total-PI3K and p-Akt/Total-Akt, as well as CCND1, while downregulating p21 and p53 expression (*P* < 0.05), indicating that IGF1R overexpression can activate the PI3K/Akt signaling pathway (Figure 8B). Overexpression of chi-miR-423-3p inhibited IRS1, p-PI3K/Total-PI3K, and p-Akt/Total-Akt, and upregulated p21 and p53, suggesting that chi-miR-423-3p may negatively regulate the PI3K/Akt pathway through IGF1R. Co-overexpression of IGF1R partially reversed the inhibitory effects of chi-miR-423-3p on the PI3K/Akt signaling pathway (*P* < 0.05).

### 3.9 Regulation of mammary epithelial cell proliferation and cell cycle progression by chi-miR-423-3p via suppression of IGF1R expression

**FIGURE 9:**
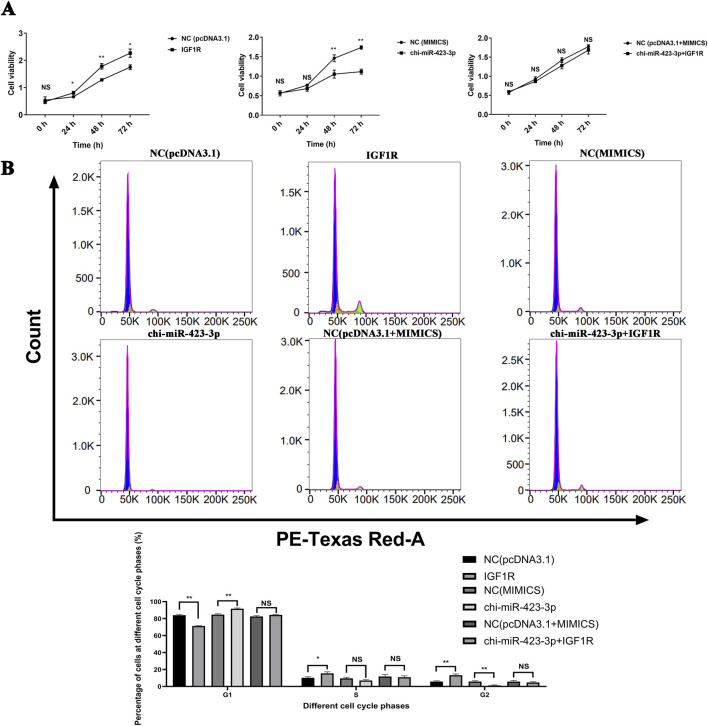
chi-miR-423-3p inhibits cell proliferation and induces G1 phase arrest by targeting IGF1R. **(A)** chi-miR-423-3p inhibits cell viability, which is rescued by IGF1R overexpression. Cell viability was assessed by CCK-8 assay at 0, 24, 48, and 72 h post-transfection. Overexpression of IGF1R promoted cell viability, while chi-miR-423-3p suppressed it. IGF1R overexpression reversed the inhibitory effect of chi-miR-423-3p. **(B)** chi-miR-423-3p induces G1 phase cell cycle arrest via targeting IGF1R. Cell cycle distribution was analyzed by flow cytometry. chi-miR-423-3p increased the proportion of cells in G1 phase and decreased the proportion in G2 phase, indicating G1 phase arrest. These effects were reversed by IGF1R overexpression. Data are presented as mean ± SEM (n = 3). *P* < 0.05 (*), *P* < 0.01 (**), NS: not significant.

CCK-8 cell proliferation assay results showed that (Figure 9A) overexpression of IGF1R significantly enhanced cell viability. Compared to the control group (NC (pcDNA3.1)), the cell proliferation rates in the IGF1R overexpression group were significantly increased by 38.97% ± 9.62% at 48 h and 29.35% ± 3.32% at 72 h (*P* < 0.01). In contrast, overexpression of chi-miR-423-3p markedly inhibited cell proliferation, with cell viability significantly decreased by 27.91% ± 5.57% at 48 h and 35.63% ± 4.04% at 72 h compared to the NC(MIMICS) group (*P* < 0.01). However, IGF1R overexpression reversed the chi-miR-423-3p-mediated inhibition of cell proliferation. There was no significant difference in cell viability between the chi-miR-423-3p + IGF1R group and the NC (pcDNA3.1+MIMICS) group, and the proliferation level was restored to near control levels.

Flow cytometry analysis of the cell cycle showed that IGF1R overexpression promoted cell cycle progression from G1 phase to G2 phase (Figure 9B). Compared to the control group (NC (pcDNA3.1)), the proportion of cells in G1 phase significantly decreased, while the proportion in G2 phase significantly increased in the IGF1R overexpression group (*P* < 0.05). Conversely, overexpression of chi-miR-423-3p led to a significant increase in the G1 phase cell population and a decrease in the G2 phase population (*P* < 0.01), suggesting that chi-miR-423-3p may inhibit cell cycle progression via IGF1R-mediated signaling. Furthermore, IGF1R overexpression counteracted the G1 phase arrest induced by chi-miR-423-3p, with no significant differences observed in the proportions of cells in G1, S, and G2 phases between the chi-miR-423-3p + IGF1R group and the NC (pcDNA3.1 + MIMICS) group.

### 3.10 chi-miR-423-3p regulates apoptosis of mammary epithelial cells by targeting IGF1R

**FIGURE 10:**
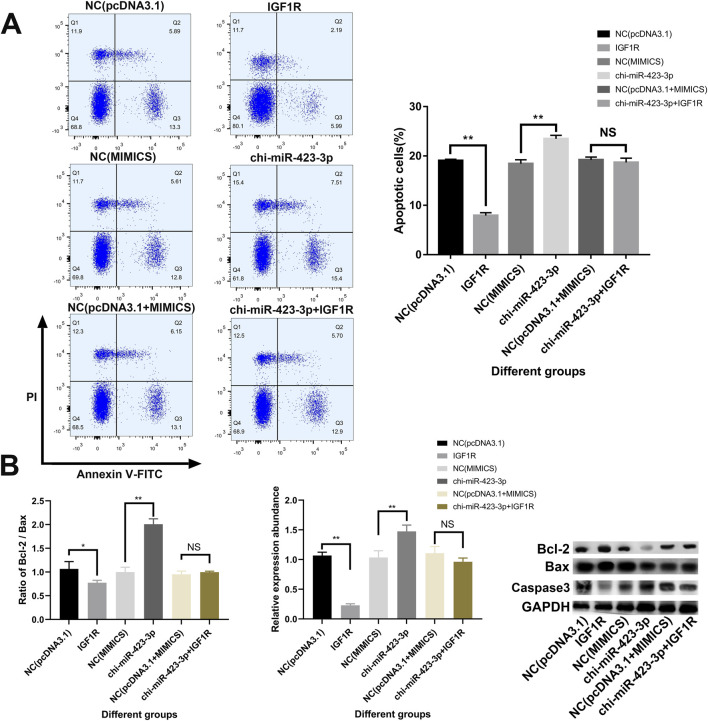
chi-miR-423-3p promotes apoptosis in goat mammary epithelial cells by targeting IGF1R. **(A)** Flow cytometry analysis of apoptosis in goat primary mammary epithelial cells transfected with chi-miR-423-3p mimics, IGF1R overexpression plasmid, or both. Representative dot plots are shown (left), and quantification of apoptotic cells (%) is summarized (right). Overexpression of chi-miR-423-3p significantly increased apoptosis, whereas co-expression of IGF1R reversed this effect. **(B)** Expression of apoptosis-related proteins Bcl-2, Bax, and Caspase 3 was examined by Western blot. The ratio of Bcl-2/Bax (left) and the relative expression of Caspase 3 (middle) were quantified. Chi-miR-423-3p significantly decreased the Bcl-2/Bax ratio and upregulated Caspase 3 expression, while IGF1R overexpression attenuated these effects. Representative Western blot bands are shown on the right. Data are shown as mean ± SEM from three independent experiments. *P* < 0.05 (*), *P* < 0.01 (**), NS: not significant.

As shown in Figure 10A, flow cytometry analysis of apoptosis revealed that overexpression of IGF1R significantly reduced the apoptosis rate compared to the control group NC (pcDNA3.1) (*P* < 0.01). In contrast, the apoptosis rate in the chi-miR-423-3p group was significantly higher than that in the NC (MIMICS) control group (*P* < 0.01). Co-transfection with chi-miR-423-3p and the IGF1R plasmid did not significantly alter the apoptosis rate compared to chi-miR-423-3p alone (*P* > 0.05).

Furthermore, Western blot analysis of apoptosis-related protein expression (Figure 10B) showed that IGF1R overexpression significantly increased the Bcl-2/Bax ratio and decreased Caspase-3 protein levels (*P* < 0.05 or *P* < 0.01). In contrast, the chi-miR-423-3p group exhibited the opposite trend, with a significant decrease in the Bcl-2/Bax ratio and a significant increase in Caspase-3 expression (*P* < 0.01). However, co-transfection of chi-miR-423-3p and the IGF1R plasmid did not result in significant changes in the Bcl-2/Bax ratio or Caspase-3 expression levels compared to chi-miR-423-3p alone (*P* > 0.05). These results suggest that chi-miR-423-3p may promote apoptosis by targeting and downregulating IGF1R expression, and that IGF1R overexpression can partially reverse the pro-apoptotic effects of chi-miR-423-3p.

## 4 Discussion

In this study, transcriptome sequencing of mammary gland tissue from dairy goats was performed to systematically analyze the expression profiles and dynamic changes of miRNAs across different physiological stages, including late gestation, early lactation, peak lactation, late lactation, and the dry period. The results revealed the critical regulatory roles of specific miRNAs in the initiation of lactation, maintenance of mammary function, involution, and structural remodeling of the mammary gland.

A total of 1,120 miRNAs were identified from the sequencing data, including 712 newly predicted miRNAs, accounting for a substantial proportion (Figure 1B). Previous studies have identified 568 conserved miRNAs and 381 potential novel miRNAs in the mammary glands of Guanzhong dairy goats during colostrum and peak lactation stages (Hou et al., 2017). Another study identified 766 miRNAs in the mammary glands of Saanen dairy goats during the involution stage, including 546 known miRNAs and 220 novel ones (Jiang et al., 2022). These findings suggest that a large number of previously uncharacterized miRNAs are involved in the regulation of mammary gland function in dairy goats, warranting further in-depth investigation.

PCA results (Figure 2A) revealed a clear clustering pattern of miRNA expression profiles across different stages of mammary gland development, indicating that miRNA expression exhibits significant stage specificity. Among the 383 differentially expressed miRNAs identified, the largest number of DE-miRNAs (221) was observed between the dry period and late gestation (Figure 2B). This finding is consistent with previous reports in Saanen dairy goat mammary tissue and further supports the notion that the dry period is a key stage in which miRNA-mediated regulation is highly active during mammary gland involution and remodeling (Jiang et al., 2022). Several miRNAs, including miR-148a-3p, miR-30f-3p, miR-381, miR-1, miR-3432-5p, and miR-708-5p, were significantly differentially expressed (Supplementary Table S4). Notably, chi-miR-148a-3p and chi-miR-30f-3p were significantly downregulated during the dry period and markedly upregulated during late gestation, with fold changes exceeding 256. miR-148a-3p is known to be abundant in milk-derived miRNAs, especially in colostrum, and is considered beneficial to neonates, as demonstrated in colostrum from dairy goats (Chen et al., 2017), cattle (Chen et al., 2010), and humans (Chiba et al., 2022). Previous studies have shown that members of the miR-148 family can regulate apoptosis (Okumura et al., 2021) and lipid metabolism (Amin Mohedin et al., 2024), suggesting a potential role in apoptosis and structural remodeling during mammary gland involution. Additionally, the miR-30 family has been implicated in the regulation of mammary epithelial cell proliferation (Yang et al., 2017), indicating its possible involvement in the regulation of mammary gland remodeling.

The UpSet plot (Figure 2C) and heatmap (Figure 2D) in this study further clarified the distribution patterns of stage-specific miRNAs. Clustering analysis (Figure 3; Supplementary Table S6) revealed six differentially expressed miRNA clusters with distinct expression profiles. Among them, miRNAs in Cluster 1 and Cluster 5 were highly expressed during the dry period, particularly miR-99a-5p, miR-206, and the newly predicted miRNAs NC_030828.1_27139 and NC_030820.1_17538, which were upregulated more than 16-fold during the dry period. Functional enrichment analysis of their target genes showed significant enrichment in pathways such as the TCA cycle, fatty acid metabolism, and steroid biosynthesis. miR-99a-5p has been reported to be associated with steroid metabolism (Li et al., 2018). miR-206 can target glucose-6-phosphate dehydrogenase, affecting the pentose phosphate pathway and thereby regulating lipid accumulation and cell proliferation (Wang et al., 2021). These findings suggest that miRNAs specifically expressed during the dry period may promote mammary epithelial cell apoptosis, lipid degradation, and energy remodeling by regulating energy metabolism, lipid synthesis, and amino acid metabolism, thus preparing the mammary gland for the next lactation cycle.

Cluster 3 was specifically highly expressed during early lactation, and its target genes were significantly enriched in autophagy and AMPK signaling pathways. Notably, miR-223-5p and miR-708-5p were markedly upregulated during this period. Previous studies have indicated that miR-223 can regulate intracellular homeostasis through autophagy (Zhou et al., 2019; Ma et al., 2024), thereby affecting the secretory function of mammary epithelial cells. In addition, miR-223-3p has been reported to alleviate myocardial oxidative stress and programmed cell death by targeting the AMPK pathway (Zhang et al., 2021). Our findings also support the notion that miR-223 may promote the functional adaptation of mammary epithelial cells to high metabolic demands in early lactation by modulating the AMPK and autophagy pathways. miR-708 has been shown to regulate multiple pathways, including AMPK signaling (Zhang et al., 2024), the PI3K/Akt pathway (Wang et al., 2020), and the Wnt/β-catenin pathway (Yu et al., 2020), thereby influencing cell proliferation. Therefore, the high expression of miR-708-5p in early lactation may contribute to the proliferation and metabolic adaptation of mammary epithelial cells, ensuring the dynamic balance of mammary gland function and improving lactation efficiency.

miRNAs in Cluster 4 and Cluster 6 were highly expressed during late gestation, and their target genes were significantly enriched in pathways such as cell adhesion, the PI3K-Akt signaling pathway, and ECM-receptor interaction. For example, miR-381 and miR-30f-5p were markedly upregulated during this stage, suggesting their potential involvement in the maturation of mammary alveolar structures and the establishment of epithelial cell polarity. Consistent with previous studies, both miR-381 and members of the miR-30 family have been shown to regulate the PI3K/Akt pathway, thereby influencing cell proliferation and structural development (Xuan et al., 2019; Varga et al., 2022). miR-381-3p has also been reported to affect mammary cancer cell growth by modulating epithelial-mesenchymal transition (EMT) (Yu et al., 2021). Additionally, miR-30 has been demonstrated to participate in the regulation of various cellular processes, including proliferation and apoptosis (Zhang et al., 2016; Fan et al., 2020; Shi et al., 2021). These findings further support the proposed regulatory roles of these miRNAs in the maturation of mammary alveolar structures.

Notably, this study also constructed a miRNA–mRNA regulatory network related to mammary gland development and identified *IGF1R*, *ESR1*, *SRC*, *PIK3CA*, and *AKT1* as hub regulatory genes (Figure 5). *ESR1* plays a crucial role in mammary gland development, remodeling, and in the regulation of cell proliferation and differentiation (Tucker et al., 2016; Will et al., 2023). During late gestation, estrogen levels rise rapidly and, in coordination with prolactin (PRL) and insulin-like growth factor 1 (IGF-1), promote the proliferation of mammary epithelial cells while inhibiting milk secretion, thereby ensuring proper structural development of the mammary gland in preparation for lactation (Gorton et al., 2009; Leonel et al., 2017). After parturition, the abrupt decline in estrogen levels due to placental expulsion facilitates the action of lactogenic hormones such as PRL and insulin (Ji et al., 2019a). This corresponds with our findings showing high ESR1 expression in late gestation and low expression in early lactation. Furthermore, this study predicted that chi-miR-200b, chi-miR-543-3p, and chi-miR-2404 may regulate ESR1 expression, suggesting that miRNAs may play key roles in the regulation of mammary gland development and lactation.

This study found that among the core genes associated with mammary gland development, IGF1R, a key node in the PI3K/Akt signaling pathway, was specifically highly expressed during the dry period (Figure 6C). IGF1R is an important receptor involved in mammary gland development, and the insulin-like growth factor (IGF) signaling pathway plays a critical role in the process of mammary gland involution (Farrelly et al., 1999; Fleming et al., 2007). IGF-1 exerts mitogenic effects through its receptor IGF1R, promoting the proliferation of mammary epithelial cells (Tamimi et al., 2011). The survival of mammary epithelial cells is regulated by signals mediated by the basement membrane and hormones such as insulin, IGF-1, and IGF-2. In bovine mammary tissue, IGF-1 is upregulated during mammary involution, a change closely related to increased rates of cellular turnover (Plath-Gabler et al., 2001). In fact, IGF-1 mRNA levels are highest during mammogenesis, decrease during lactation and galactopoiesis, and rise again during involution (Sorensen et al., 2006). Meanwhile, the sensitivity of mammary tissue to IGF-1 increases, as IGF1R expression is significantly elevated in the mammary glands of cows after drying off. These findings suggest that during mammary gland involution, IGF1R may play a regulatory role at specific stages by enhancing IGF-1-mediated signaling, indicating that IGF1R has important biological functions in the mammary gland during the dry period.

In recent years, miR-423 has been frequently reported to be closely associated with the abnormal proliferation of breast cancer cells (Zhao et al., 2015; Dai et al., 2020; Morales-Pison et al., 2021). In this study, chi-miR-423-3p was found to be lowly expressed during the dry period (Figure 6C). Experimental validation showed that chi-miR-423-3p significantly downregulates the expression of IGF1R, and dual-luciferase reporter assays further confirmed a direct targeting relationship between chi-miR-423-3p and IGF1R (Figure 7). Moreover, functional assays clearly demonstrated that chi-miR-423-3p suppresses mammary epithelial cell proliferation by inhibiting IGF1R expression, induces G1 phase cell cycle arrest, promotes apoptosis, and inhibits activation of the PI3K/Akt signaling pathway (Figures 8–10). These findings indicate that chi-miR-423-3p plays an important regulatory role in mammary gland involution and remodeling during the dry period. Due to its significantly reduced expression during this stage, the suppressive effect of miR-423-3p on IGF1R is alleviated, thereby enhancing the activity of the IGF1R–PI3K/Akt signaling pathway. This promotes the survival and proliferation of mammary epithelial cells, accelerating the remodeling of mammary tissue during the dry period.

This study systematically examined the role of the chi-miR-423-3p/*IGF1R* regulatory axis in mammary gland development by integrating 15 transcriptome datasets from goat mammary tissues and conducting functional validation using primary mammary epithelial cells. Nonetheless, several limitations should be acknowledged. The sample size for each developmental stage was limited to three biological replicates, which may reduce statistical power and constrain the generalizability of the findings. Previous studies have recommended the inclusion of at least five transcriptome replicates to ensure adequate testing power (Yu L. et al., 2017). Additionally, transcriptome data were derived from two independent experimental batches, and differences in sampling time and sequencing platforms may have introduced batch effects (Kukurba and Montgomery, 2015). Although efforts were made to collect tissues from consistent anatomical sites, the inherent structural complexity and cellular heterogeneity of the mammary gland could have influenced transcriptomic outcomes (Cui et al., 2014). Another limitation is that miRNA target prediction was performed using only the miRanda algorithm, which may lead to false positives or missed targets; incorporating multiple algorithms such as TargetScan, RNAhybrid, and miRWalk, along with experimental validation, would improve accuracy (Agarwal et al., 2015; Khan et al., 2015). In this study, we preliminarily validated the interaction between miR-423-3p and *IGF1R* through dual-luciferase reporter assays; however, high-throughput methods such as RIP-seq or CLIP-seq are recommended for future studies to systematically verify miRNA–mRNA interactions. Furthermore, current functional assays were based on *in vitro* culture models that may not fully reflect the *in vivo* mammary microenvironment. Future research should consider increasing the number of biological replicates, standardizing sampling protocols, employing uniform sequencing platforms, and performing *in vivo* validation using model organisms. Integrative approaches combining single-cell transcriptomics and multi-omics analyses are also encouraged to uncover additional regulatory miRNAs and downstream targets involved in mammary gland development and remodeling.

## 5 Conclusion

In summary, this study systematically revealed the dynamic expression patterns of miRNAs in the mammary tissue of dairy goats across different physiological stages and clarified the key role of the miR-423-3p/IGF1R regulatory axis in mammary gland remodeling. Future research may further explore the targets and mechanisms of more stage-specific miRNAs and validate the functions of newly predicted miRNAs, aiming to comprehensively uncover the molecular mechanisms underlying mammary gland remodeling. These findings will provide important theoretical support for improving mammary health, milk production performance, and reproductive efficiency in dairy goats.

## Data Availability

The datasets presented in this study can be found in online repositories. The names of the repository/repositories and accession number(s) can be found in the article/Supplementary Material.
