# Supplementary material for: Transcriptomic analysis of mammary gland tissues in lactating and non-lactating dairy goats reveals miRNA-mediated regulation of lactation, involution, and remodeling
Source: Front Cell Dev Biol. 2025 May 30;13:1604855. doi: 10.3389/fcell.2025.1604855 (PMC12162918; doi:10.3389/fcell.2025.1604855)
Supplement: Supplementary file 1 [file DataSheet1.zip › Supplementary Materials/Supplementary_Material.docx]

Supplementary Material

#
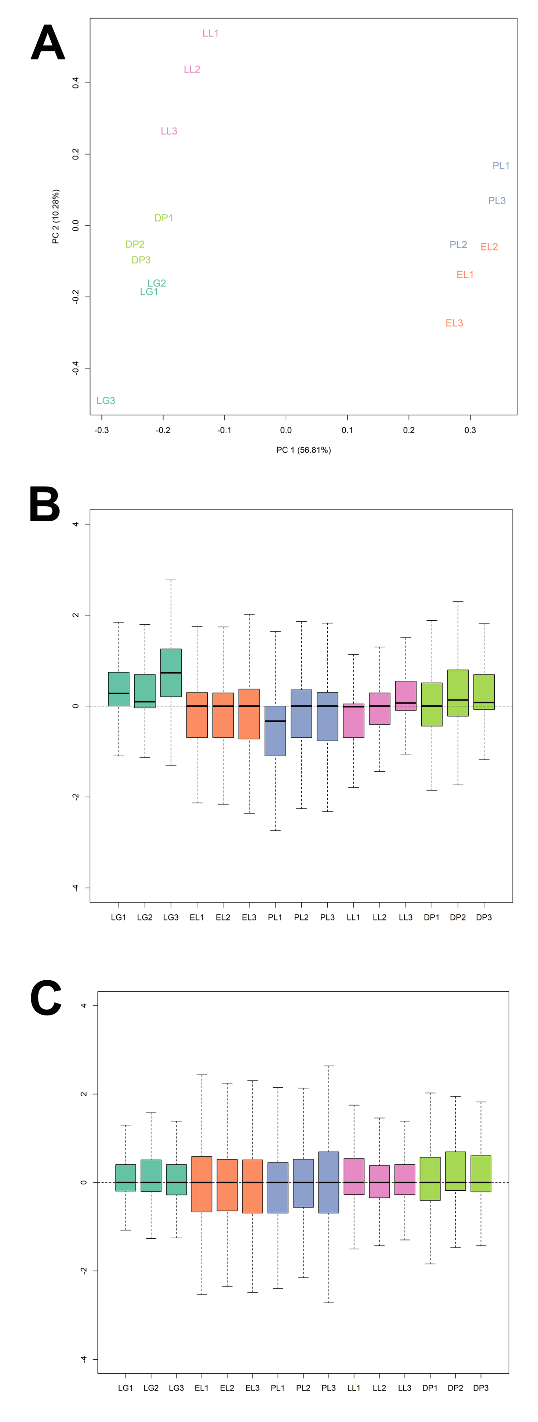
Supplementary Figures

**Supplementary Figure 1.** **Quality control and normalization assessment of transcriptome data.** **(A)** Principal component analysis (PCA) plot of all samples before RUVseq correction. **(B)** Relative log expression (RLE) plot of all samples before filtering lowly expressed genes. Some samples display notable deviations from the median, suggesting the need for further normalization. **(C)** RLE plot after filtering low-expression genes. Variations across samples are reduced and distributions are more centered around zero, indicating improved normalization quality.


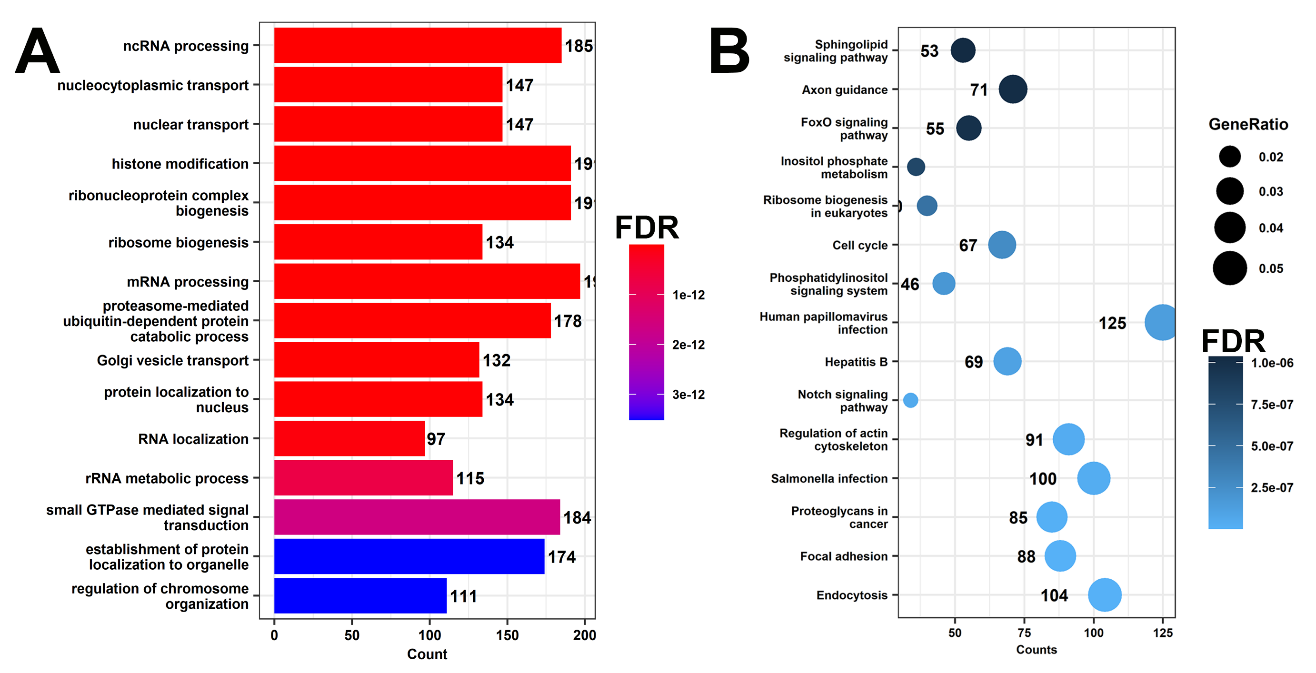


**Supplementary Figure 2. Functional enrichment analysis of target genes of differentially expressed miRNAs in Cluster 2.** **(A)** Gene Ontology (GO) enrichment analysis (Biological Process category) of target genes regulated by Cluster 2 miRNAs. The x-axis represents the number of enriched genes, and the color scale indicates the false discovery rate (FDR). **(B)** KEGG pathway enrichment analysis of target genes regulated by Cluster 2 miRNAs. Dot size represents the GeneRatio, and the color gradient corresponds to the FDR value.


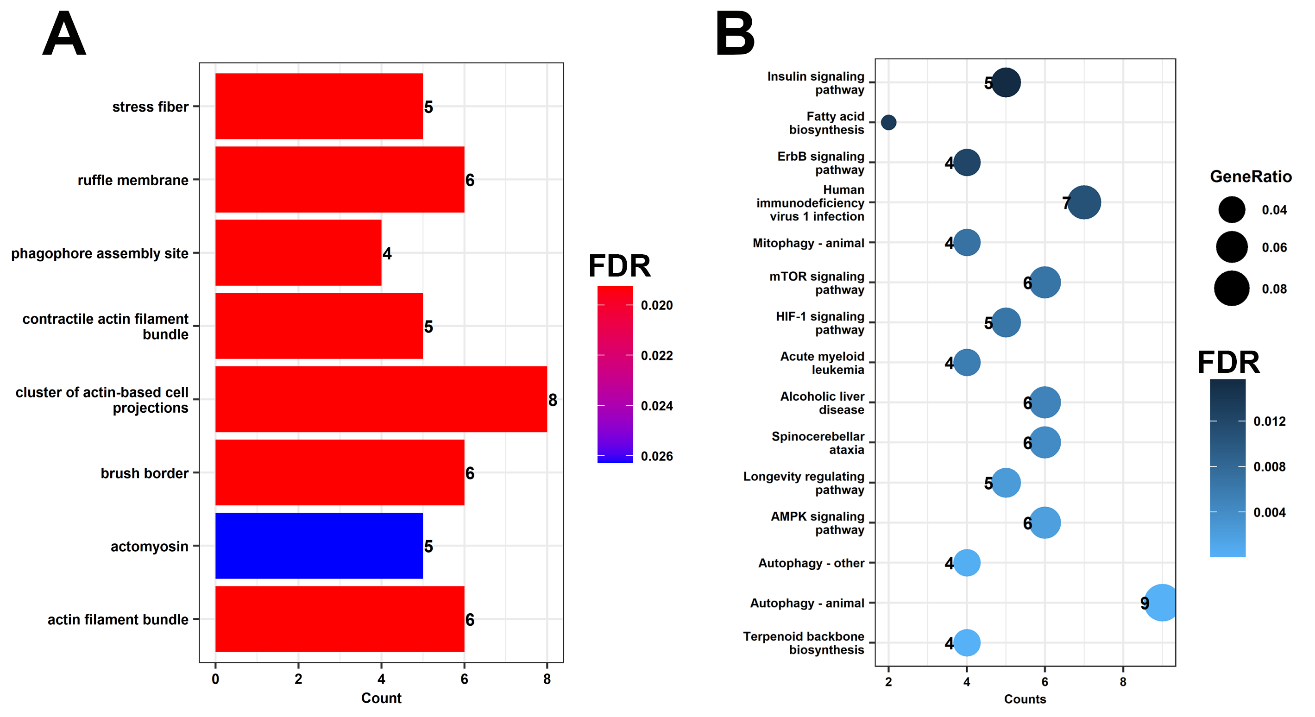


**Supplementary Figure 3. Functional enrichment analysis of target genes of differentially expressed miRNAs in Cluster 3. (A)** Gene Ontology (GO) enrichment analysis (Biological Process category) of target genes regulated by Cluster 3 miRNAs. The x-axis represents the number of enriched genes, and the color scale indicates the false discovery rate (FDR). **(B)** KEGG pathway enrichment analysis of target genes regulated by Cluster 3 miRNAs. Dot size represents the GeneRatio, and the color gradient corresponds to the FDR value.


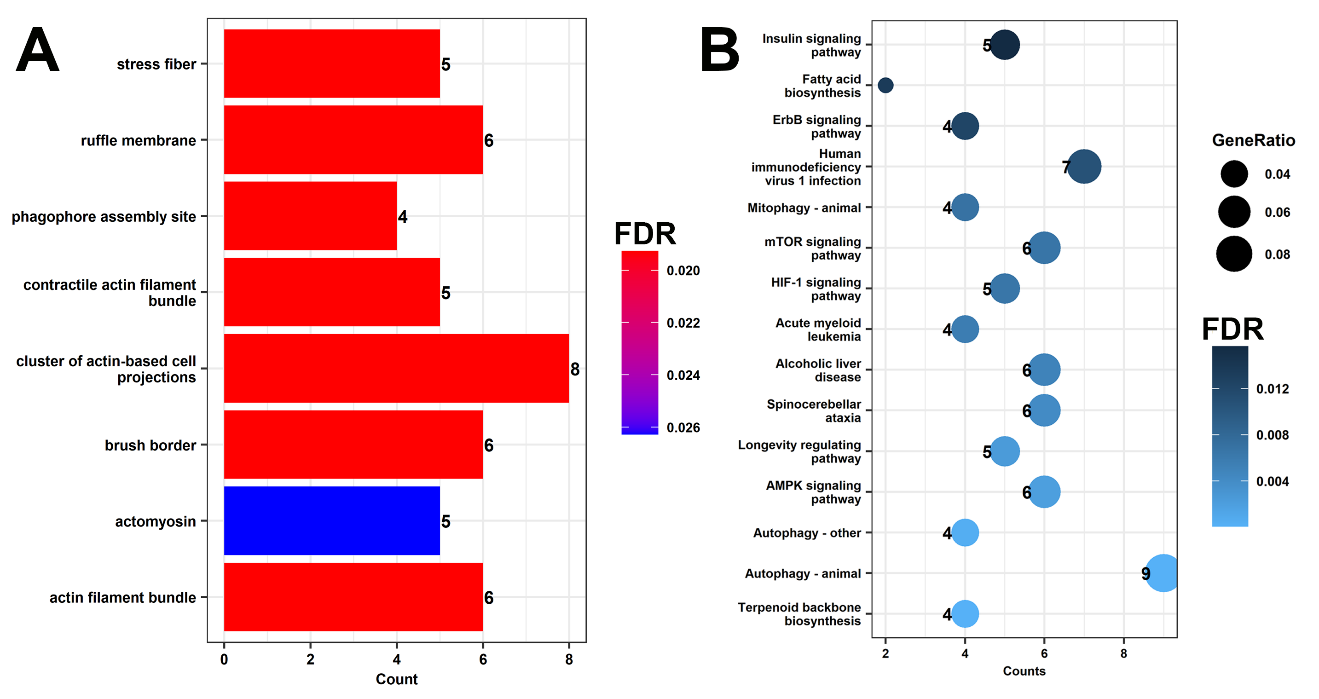


**Supplementary Figure 4. Functional enrichment analysis of target genes of differentially expressed miRNAs in Cluster 4. (A)** Gene Ontology (GO) enrichment analysis (Biological Process category) of target genes regulated by Cluster 4 miRNAs. The x-axis represents the number of enriched genes, and the color scale indicates the false discovery rate (FDR). **(B)** KEGG pathway enrichment analysis of target genes regulated by Cluster 4 miRNAs. Dot size represents the GeneRatio, and the color gradient corresponds to the FDR value.


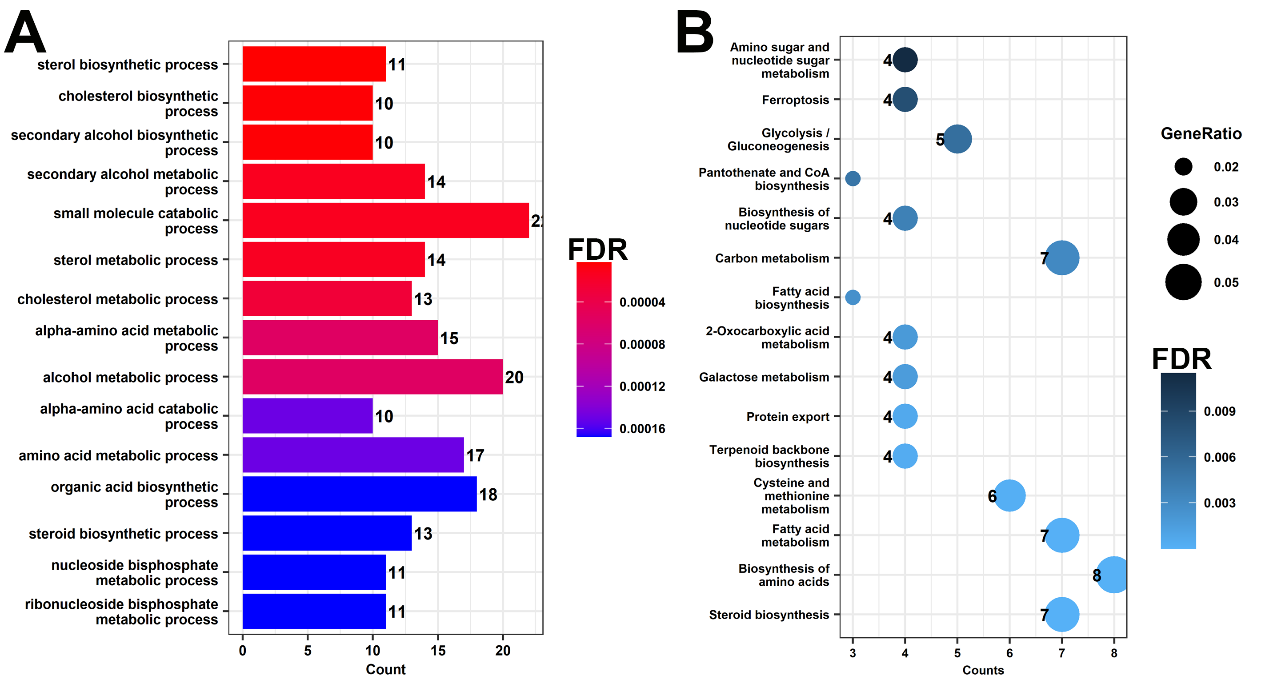


**Supplementary Figure 5. Functional enrichment analysis of target genes of differentially expressed miRNAs in Cluster 5. (A)** Gene Ontology (GO) enrichment analysis (Biological Process category) of target genes regulated by Cluster 5 miRNAs. The x-axis represents the number of enriched genes, and the color scale indicates the false discovery rate (FDR). **(B)** KEGG pathway enrichment analysis of target genes regulated by Cluster 5 miRNAs. Dot size represents the GeneRatio, and the color gradient corresponds to the FDR value.


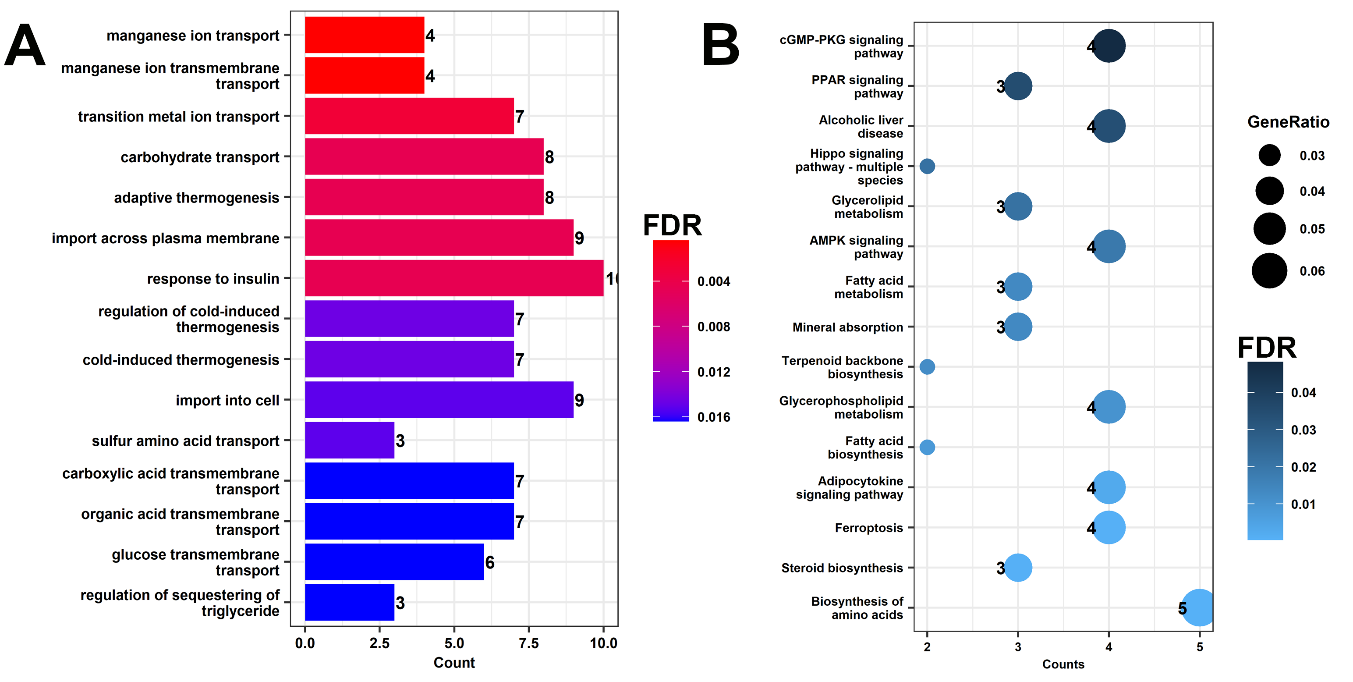


**Supplementary Figure 6. Functional enrichment analysis of target genes of differentially expressed miRNAs in Cluster 6. (A)** Gene Ontology (GO) enrichment analysis (Biological Process category) of target genes regulated by Cluster 6 miRNAs. The x-axis represents the number of enriched genes, and the color scale indicates the false discovery rate (FDR). **(B)** KEGG pathway enrichment analysis of target genes regulated by Cluster 6 miRNAs. Dot size represents the GeneRatio, and the color gradient corresponds to the FDR value.
