# Supplementary figures and images for: Transcriptomic analysis of mammary gland tissues in lactating and non-lactating dairy goats reveals miRNA-mediated regulation of lactation, involution, and remodeling
Source: Front Cell Dev Biol. 2025 May 30;13:1604855. doi: 10.3389/fcell.2025.1604855 (PMC12162918; doi:10.3389/fcell.2025.1604855)

# Figure 8A

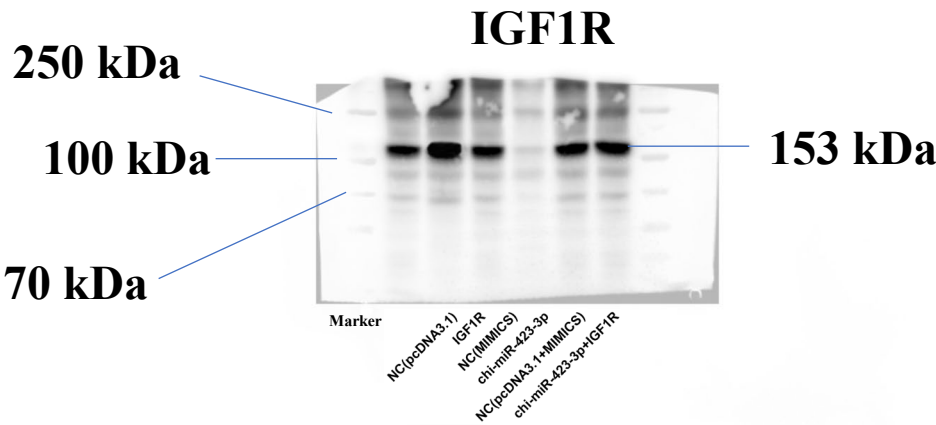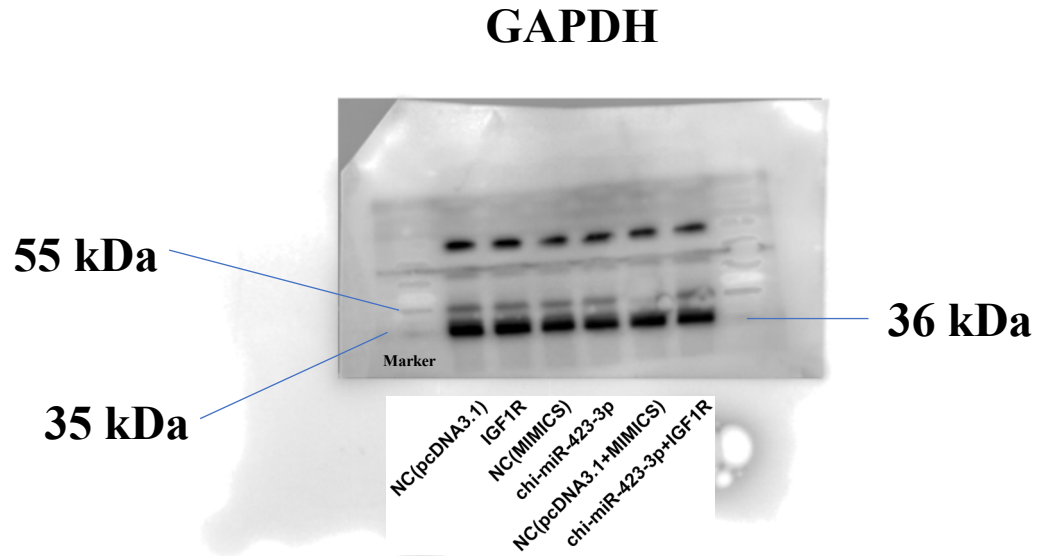

# Figure 8B

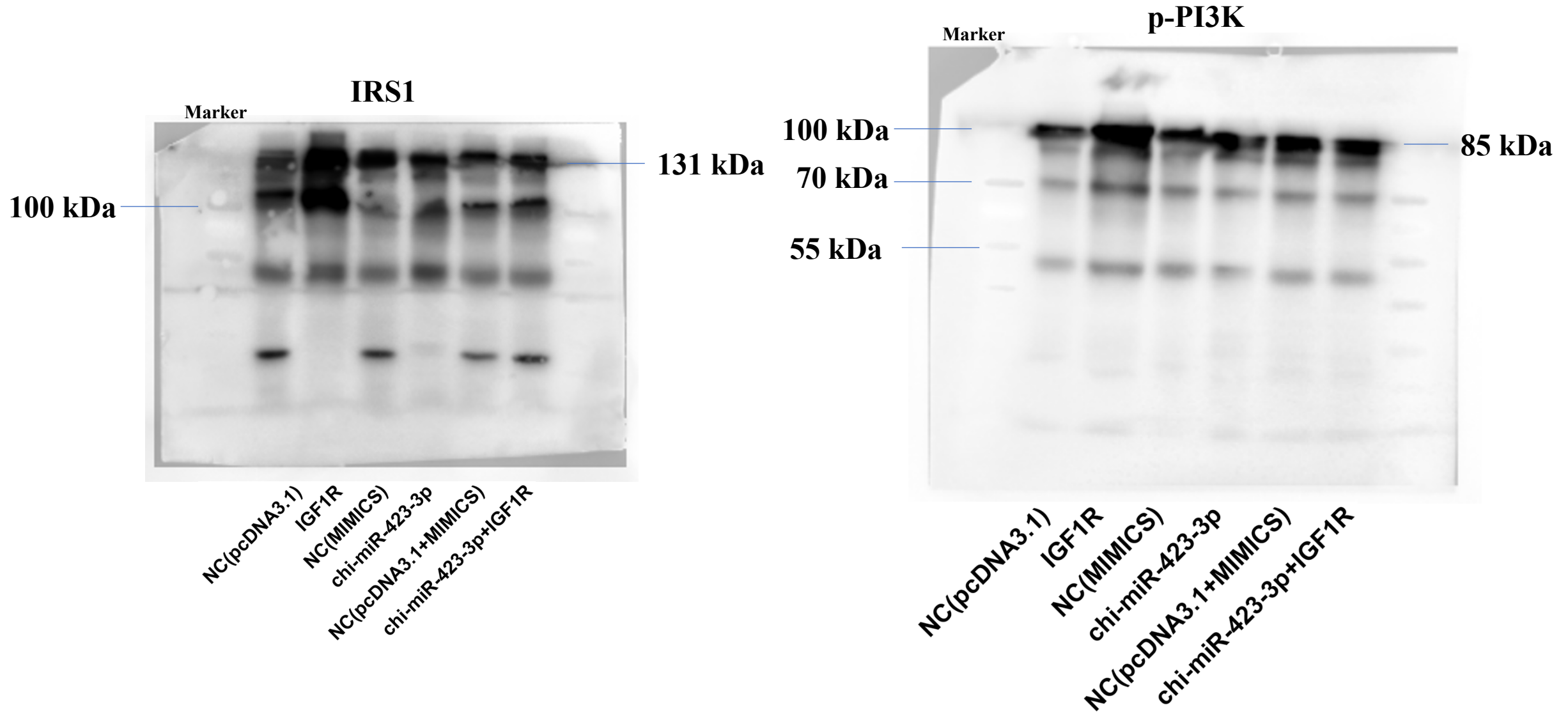

# Figure 8B

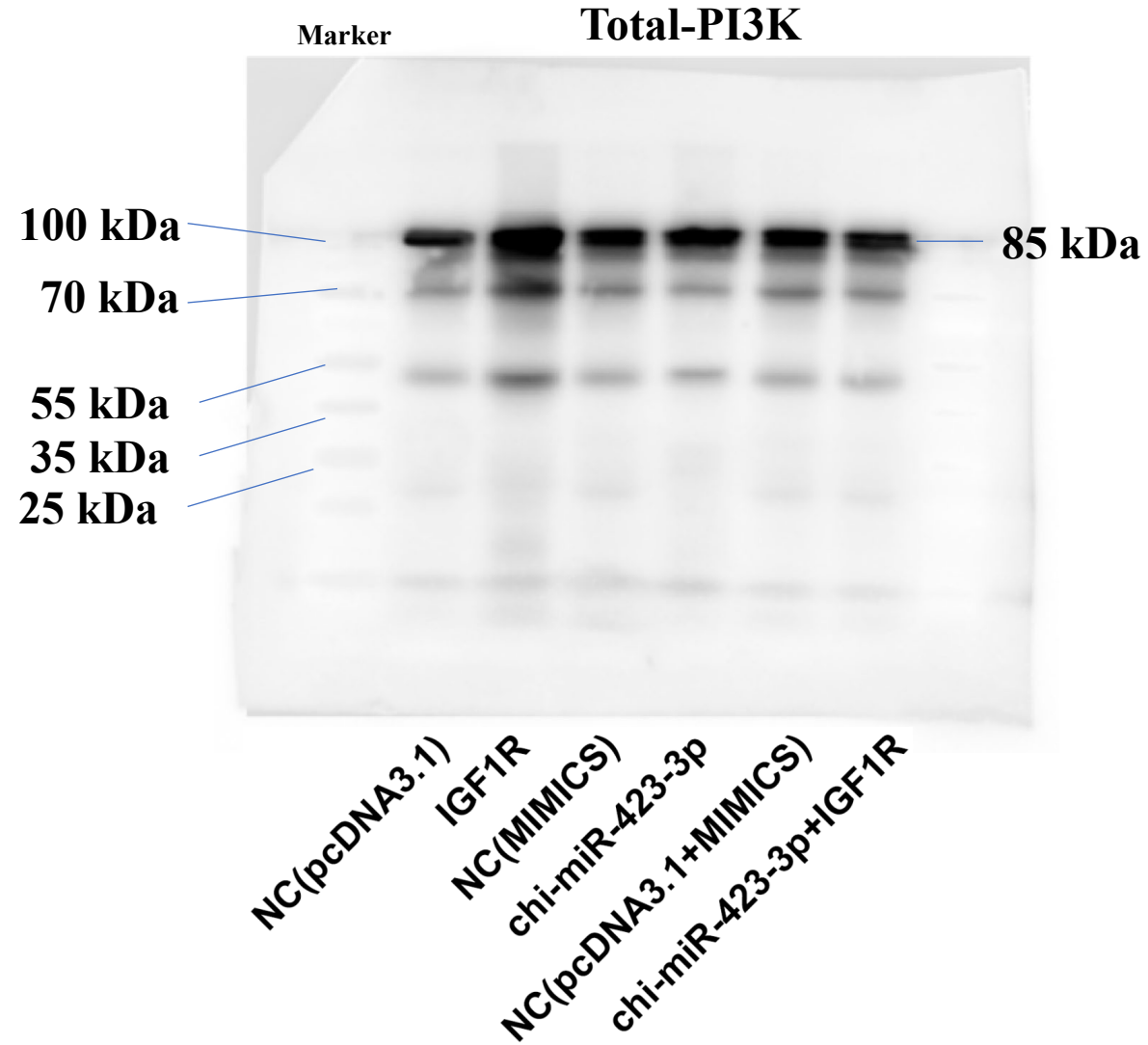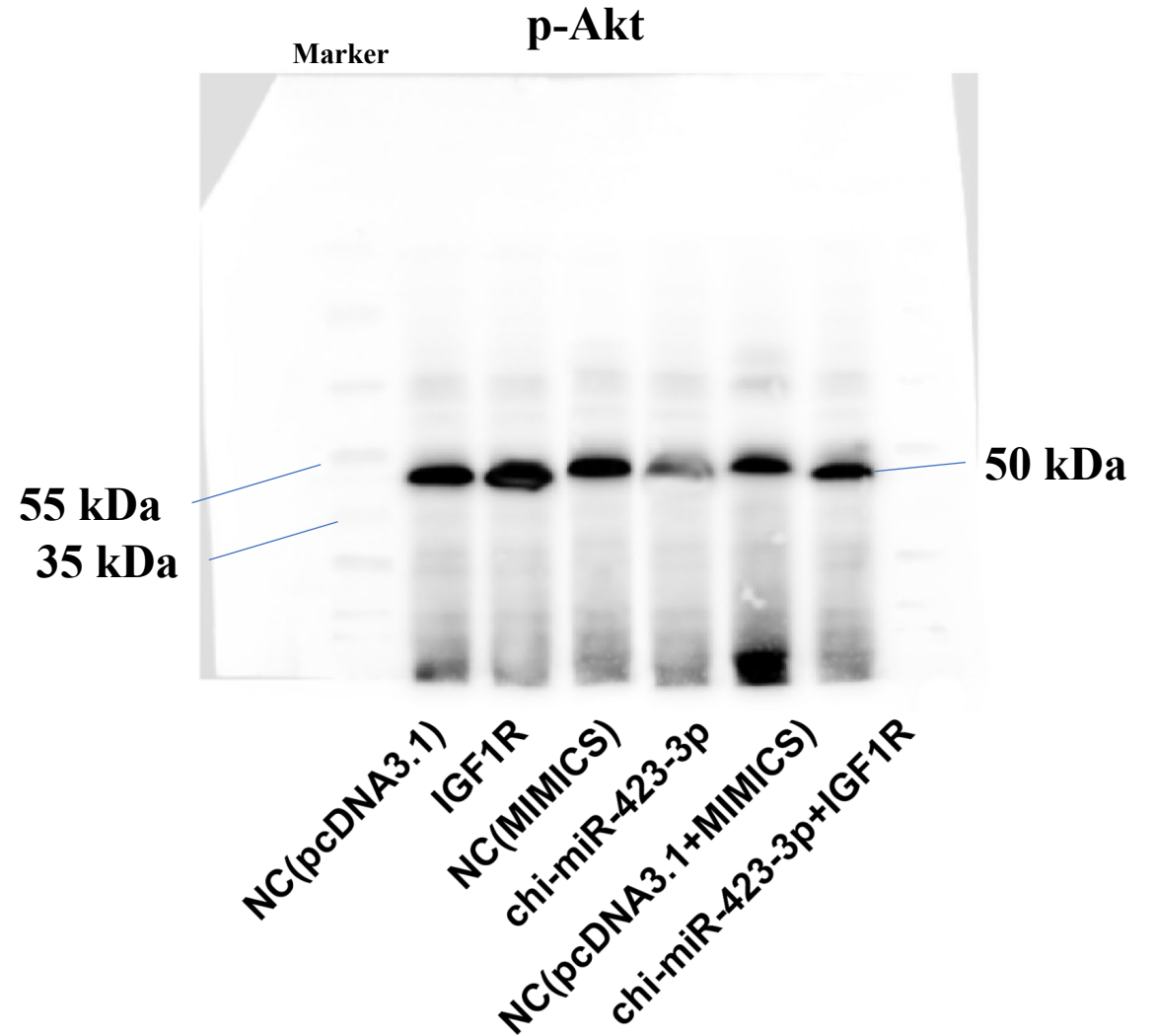

Figure 8B

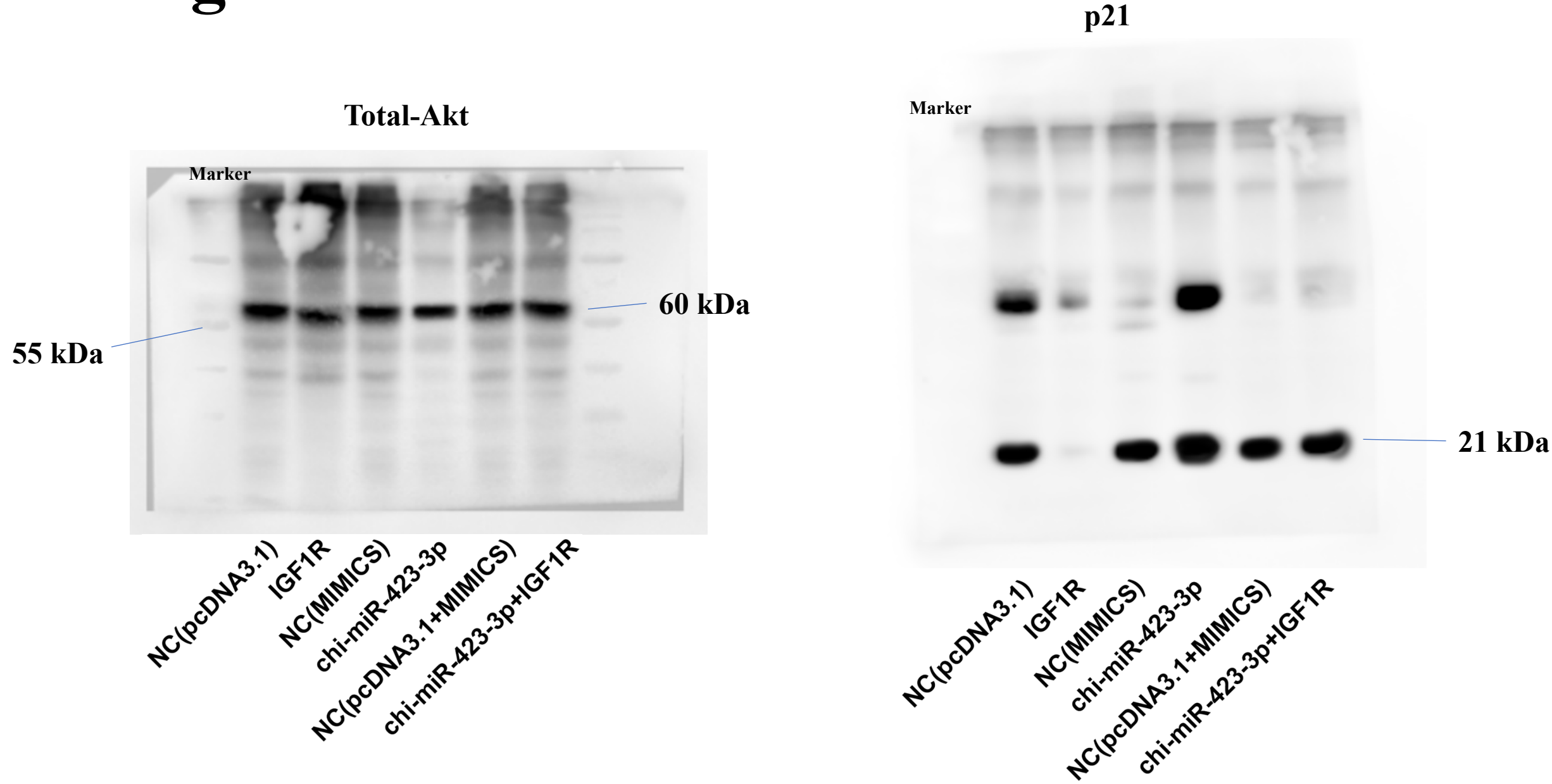

# Figure 8B

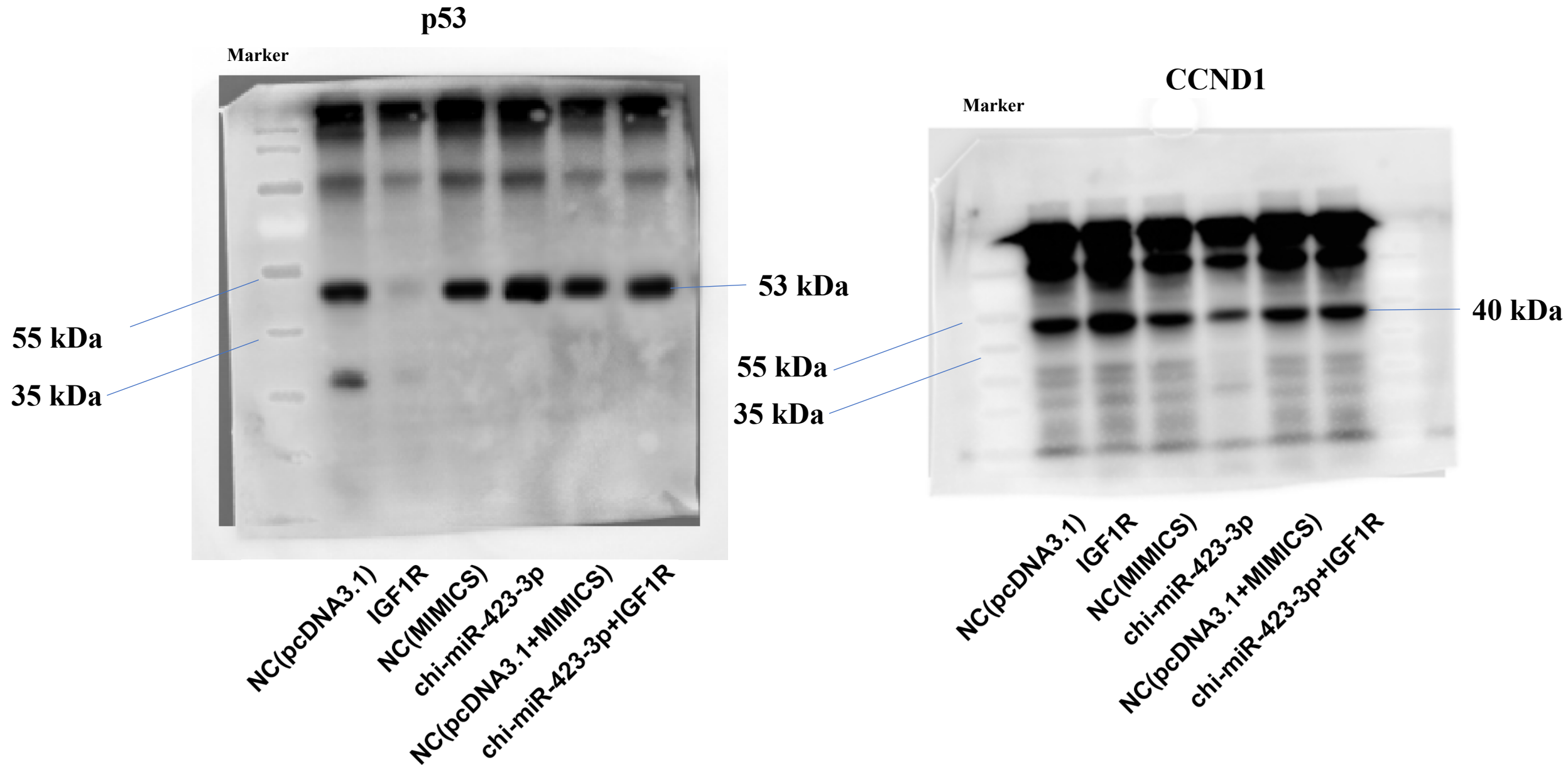

# Figure 8B

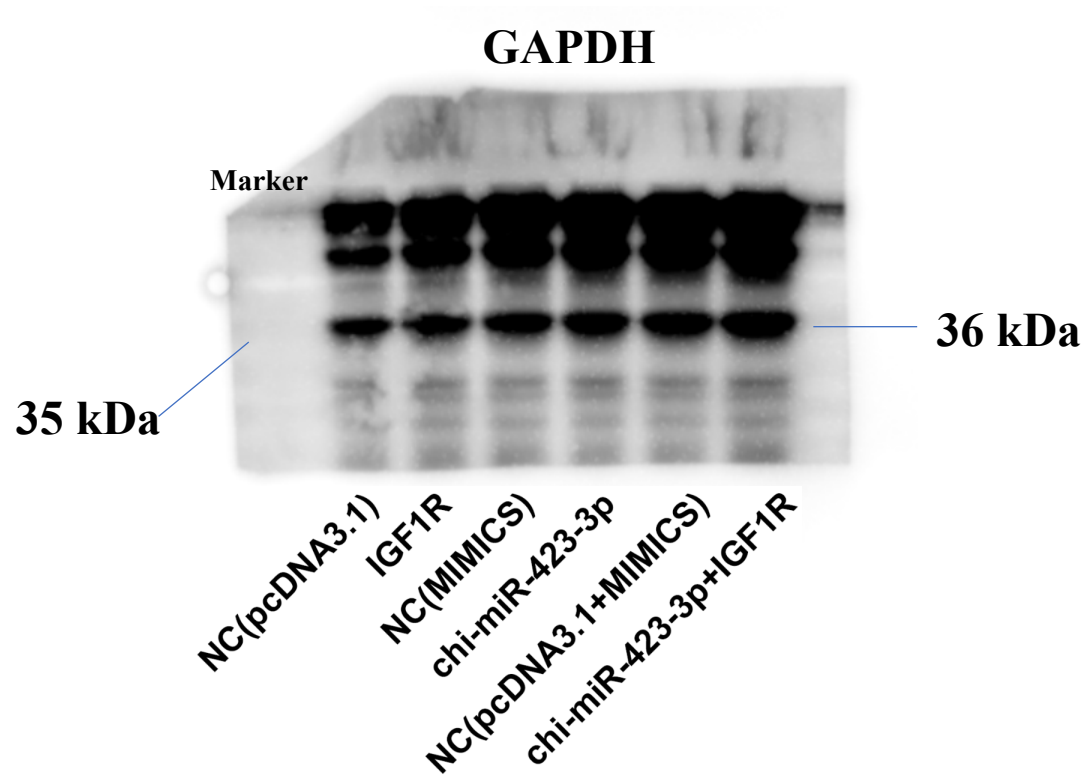

# Figure 10B

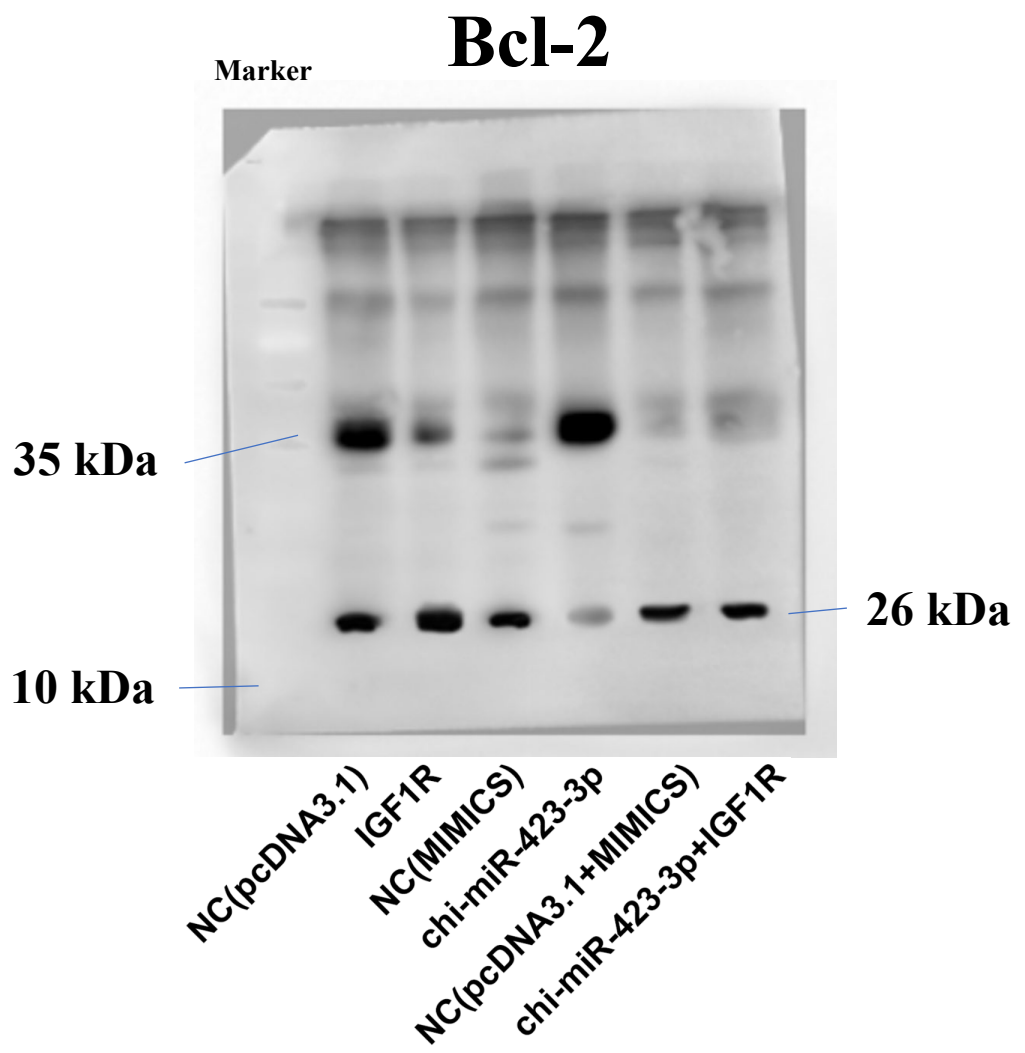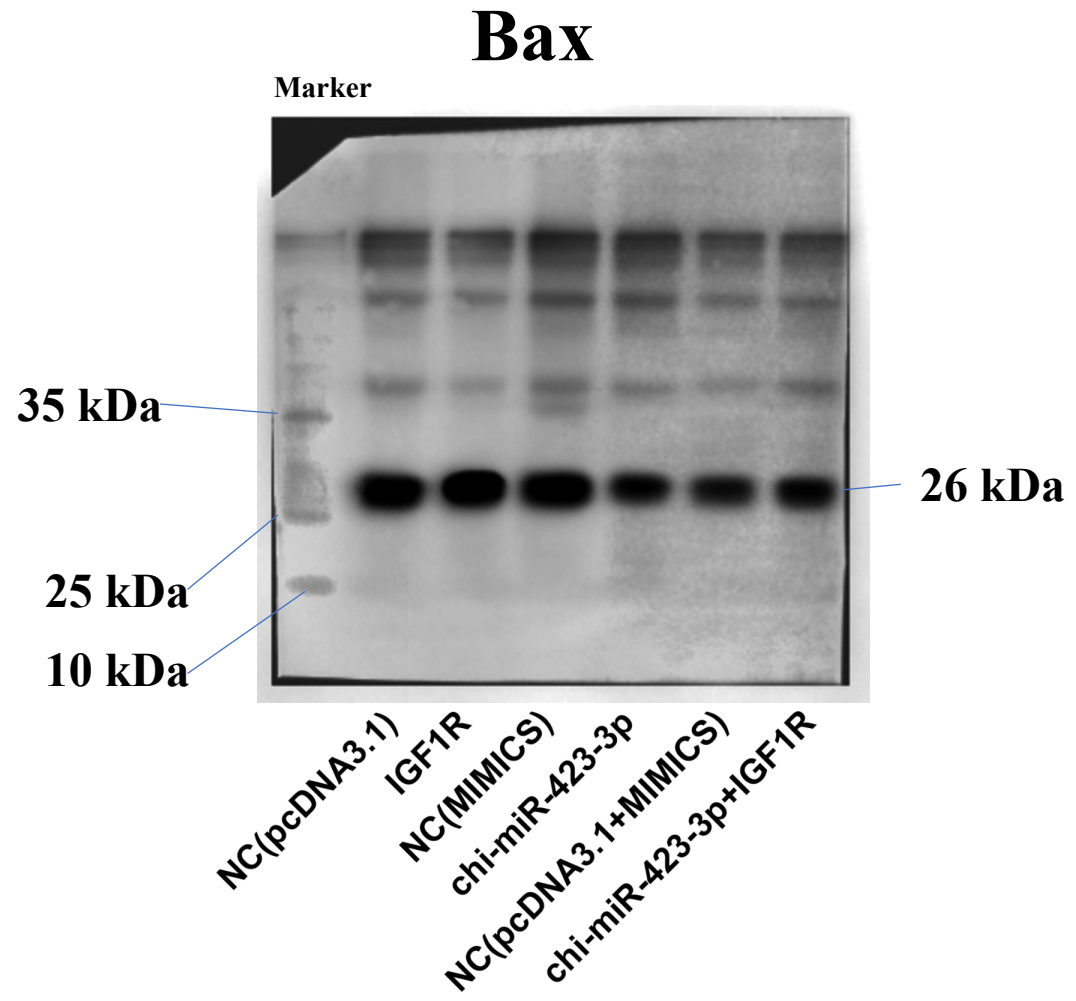

# Figure 10B

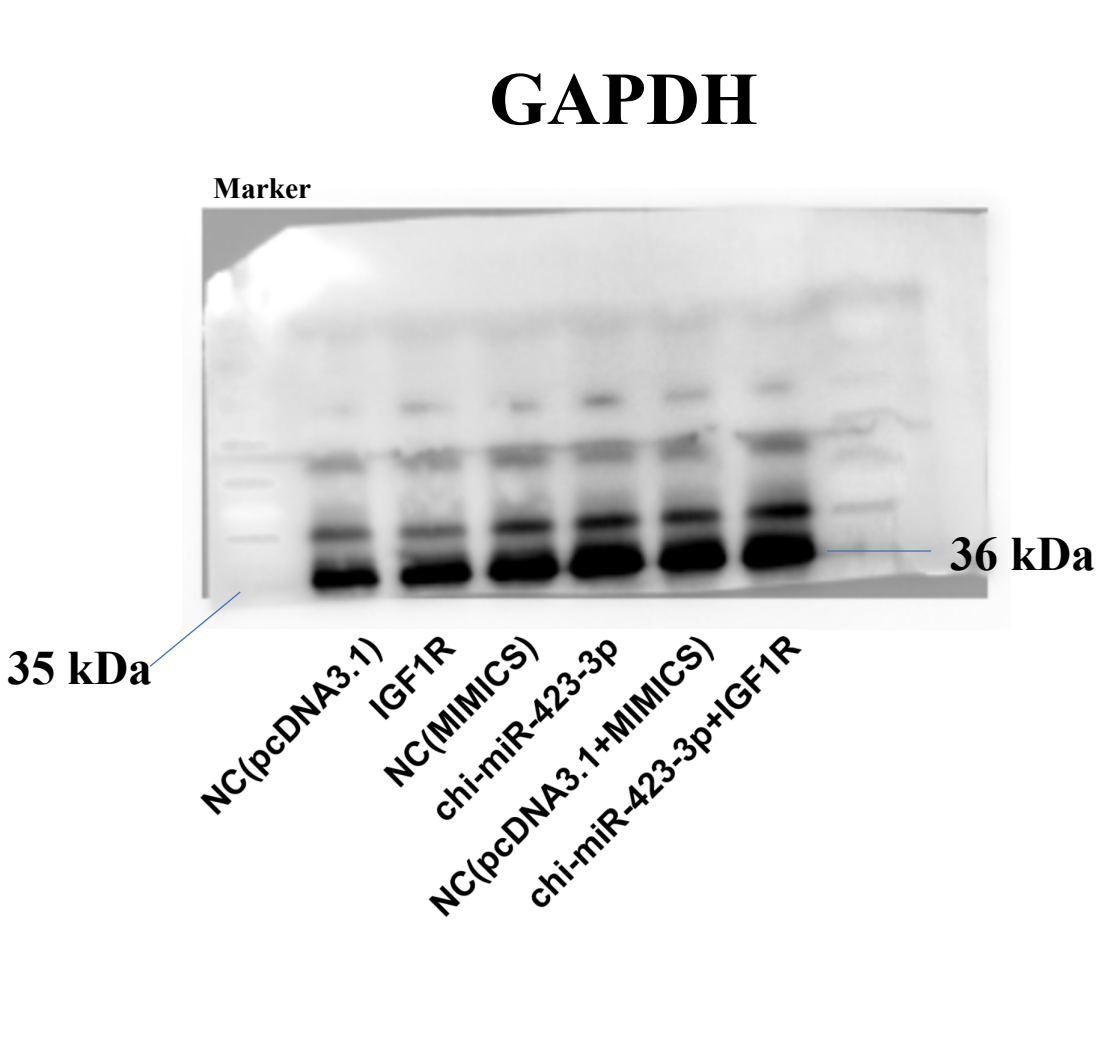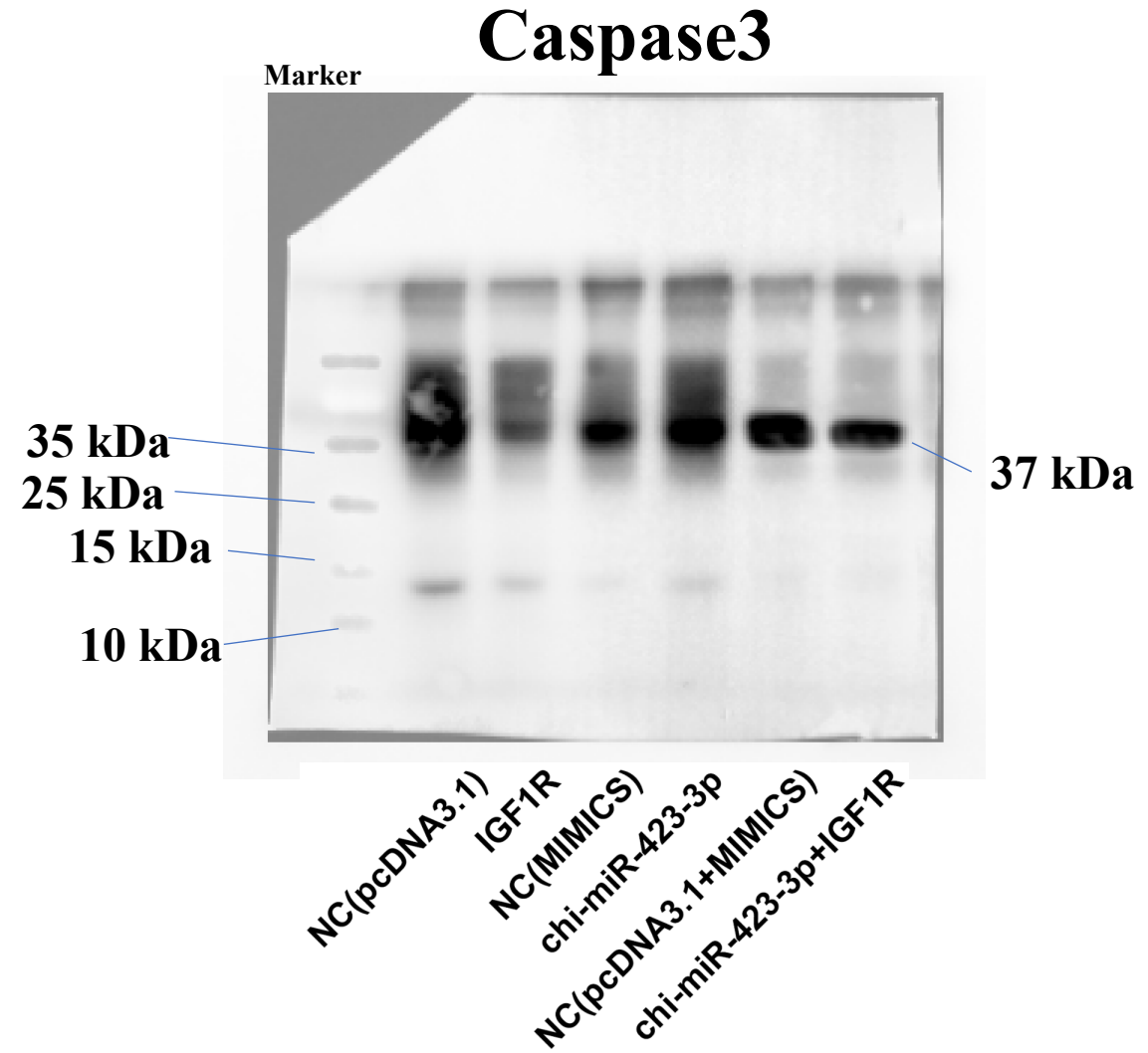

Supplement: Supplementary file 1 [file DataSheet1.zip › Supplementary Materials/Original Images for Blots.pdf]
